# Immune-enhancing activity of polysaccharides and flavonoids derived from *Phellinus igniarius* YASH1

**DOI:** 10.3389/fphar.2023.1124607

**Published:** 2023-04-25

**Authors:** Xiaoya Zhu, Ruirui Guo, Xiayu Su, Kun Shang, Conglian Tan, Junheng Ma, Yuemeng Zhang, Dan Lin, Yueran Ma, Min Zhou, Jiale Yang, Qiqi Wu, Jiale Sun, Zhuoling Wang, Yuyue Guo, Ruifan Su, Xiangyi Cui, Jiming Han, Yuhong Lü, Changwu Yue

**Affiliations:** Yan’an Key Laboratory of Microbial Drug Innovation and Transformation, School of Basic Medicine, Yan’an University, Yan’an, Shaanxi, China

**Keywords:** *Phellinus igniarius*, polysaccharides, flavonoids, antioxidants, immunocompromised, intestinal flora, SCFA

## Abstract

**Introduction:** *Phellinus igniarius (P. igniarius)* (Sanghuang) is a widely used traditional Chinese medicine fungus, and its natural products have great potential for clinical application in immune enhancement. This study aimed to explore the immune-enhancing activity and underlying mechanisms of the polysaccharides and flavonoids derived from *Phellinus igniarius* (*P. igniarius*) and to provide a theoretical and experimental basis for the development of novel drugs.

**Methods:** Wild *P. igniarius* YASH1 from the Loess Plateau in Yan’an region was collected, and polysaccharides and total flavonoids were extracted, isolated and identified from mycelium and sporophore. *In vitro* antioxidant activity was detected through the scavenging activity of hydroxyl radicals and total antioxidant capacity. Cell Counting Kit-8 and trypan blue detection kit were used to detect the effect of extract polysaccharides and flavonoids on the proliferation and phagocytosis ability of immune cells. To assess the effect of the drugs on cytokine secretion by immune cells and immune recovery in immunocompromised mice, the expression of interleukin (IL)-2, IL-6, interferon (IFN)-γ, and tumor necrosis factor (TNF)-α were examined at the cellular and animal levels. The species composition, abundance of gut microbiota and the altered content of short-chain fatty acids in the feces were analyzed to elucidate the possible mechanisms of drugs by 16S ribosomal RNA (rRNA) amplifiers sequencing and liquid chromatography-tandem mass spectrometry (LC-MS/MS).

**Results:** Both polysaccharides and flavonoids derived from mycelium or sporophore had antioxidant activity and may stimulate the expression and secretion of IL-2, IL-6, and IFN-γ in immune cells while inhibiting TNF-α expression and secretion and increasing IL-2, IL-6, and IFN- γ expression levels in mice. Furthermore, polysaccharides and flavonoids from mycelium and sporophore showed different effects on the metabolic response of intestinal short-chain fatty acids (SCFAs) in mice, and the use of these drugs remarkably changed the species composition and abundance of intestinal flora in mice.

**Discussion:** Polysaccharides and flavonoids from *P. igniarius* YASH1 mycelium and sporophore have *in vitro* antioxidant activity, and they affect the promotion of cell proliferation, stimulation of IL-2, IL-6, and IFN-γ secretion, and inhibition of TNF-α expression in immune cells. Polysaccharides and flavonoids from *P. igniarius* YASH1 may enhance immunity in immunocompromised mice and remarkably affect the intestinal flora and content of SCFAs.

## 1 Introduction

Natural products derived from medicinal fungi, such as polysaccharides or total flavonoids, have biological activities that can improve the immunity of the body, so they have great clinical application potential for immune enhancement (Chen H et al., 2016; Chugh et al., 2022). However, many studies have shown that even for the same medicinal fungi, the yield and structure of active natural products produced by them may vary greatly due to their different habitats or laboratory culture conditions (Chopraet al., 2021). In other words, habitat remarkably affects the quality of medicinal fungi, resulting in differences in the active ingredients and medicinal value of medicinal fungi depending on the environment (Jan et al., 2022).

*Phellinus igniarius (P. igniarius)* (Sanghuang) is a traditional Chinese medicinal fungus which has been widely used in traditional Chinese medicine for more than 2,000 years, and the natural products from *P. igniarius* have great potential for clinical application (Zhou et al., 2022).

Recent studies have demonstrated *P. igniarius* contains both complex, high and low molecular weight natural bioactive compounds, the majority of which are triterpenoids, polysaccharides and total flavonoids, and have important applications in antioxidant, immunity enhancement, and tumor suppression; as well as in the prevention and treatment of cirrhosis, ascites, hypoglycemia, hypolipidemia (Chen Y et al., 2016; Gao et al., 2017; Sun et al., 2018; Kim et al., 2019).

Intestinal flora plays an essential role in the body’s resistance to various diseases or pathogenic infections (Li et al., 2021). A large number of studies on polysaccharides or flavonoids from *P. igniarius* suggest that these natural products play an important role in regulating the composition and abundance of intestinal flora and improving the body’s immunity, which may have great potential for clinical application (Zapora et al., 2016; Wang et al., 2019). Intestinal flora and short-chain fatty acids (SCFAs) influence the immune cell function by influencing gene expression, cell differentiation, cell proliferation, and apoptosis (Liu et al., 2020).

In this study, wild *P. igniarius* YASH1 parasitized the wild *Sophora japonica,* which was collected around Yan’an, a typical habitat in the hilly-gully region of the Loess Plateau. Polysaccharides and total flavonoids were extracted, isolated, and identified from mycelium and sporophore, and used to provide a theoretical basis for the development and utilization of *P. igniarius* by analyzing their immune enhancement activity, effect on the composition and abundance of intestinal flora, and effect on SCFA synthesis.

## 2 Materials and methods

### 2.1 Animals, cell lines, fungi, and reagents

Kunming mice (SPF grade, 7 weeks old weighing 18–22 g) were obtained from Jiangsu Huachuang Xinnuo Pharmaceutical Technology Co., Ltd. (Jiangsu, China, SCXK (Su) 2020-0009). The mice were housed at the Animal Experiment Center of Yan’an University and given free access to food and water under temperature- and humidity-controlled conditions with a 12 h light/dark cycle. The animals were acclimatized to laboratory conditions for 7 days prior to the commencement of experimentation. All the animal experiments were approved by the Animal Ethics Committee of Yan’an University (No. 2020-009) and performed in accordance with the National Institutes of Health Guidelines for the Care and Use of Laboratory Animals. Cell lines including Raji cell (B cell), Jurkat cell (T cell) and Raw264.7 cell (mouse macrophage) were kindly donated by Prof. Ding Xiang from China West Normal University. The cell lines were preserved by the Yan’an Key Laboratory of Microbial Drug Innovation and Transformation. Fungus samples were collected from the wild locust trees on the Loess Plateau around Yan’an University. The total DNA of sporophore and mycelium was extracted and used to identify species through ITS amplification and sequencing, and the sample was named as *P. igniarius* YASH1. Biochemical reagents, molecular biology kits, and cell biology reagents for DNA exaction and amplification, cell culture, natural products extraction and identification, were obtained from Beijing Solarbio Science and Technology Co., Ltd. Primers used for species identification were synthesized by Sangon Biotech (Shanghai) Co., Ltd. Enzyme-linked immunosorbent assay (ELISA) kits for interleukin (IL)-2, IL-6, interferon (IFN)-γ, and tumor necrosis factor (TNF)-α and kits for antioxidant or hydroxyl radical scavenging capacity assay as well as Cell Counting Kit-8 for cell proliferation and cytotoxicity assays were all from Beijing Solarbio Science and Technology Co., Ltd.

### 2.2 Species identification and cultivation of *Phellinus igniarius* YASH1 mycelium

After disinfection with a chemical disinfectant, the sporophore was cut into thin slices, and the slices were inoculated onto the potato dextrose agar in a Petridish and incubated in an incubator controlled at 28 °C for 10 days. A piece of mycelium was aseptically transferred to a new potato dextrose agar Petridish and incubated at 28°C for 30 days, and the mycelium was collected to extract the natural products. The total DNA of sporophore and mycelia was extracted by guanidine isothiocyanate/phenol extraction. ITS1 (5′TCC​GTG​AAC​CTG​CGG-3′) and ITS4 (5′TCC​CGT​TAT​TGA​TAT​GC-3′) primers were used to amplify the DNA of specific sequence regions and sequenced using the Sanger method. The fungus was named *P. igniarius* YASH1 after the DNA sequence was blasted by BlastN.

### 2.3 Extraction, purification, and identification of polysaccharides and flavonoids

Polysaccharides were extracted, purified, and identified as described by Yuan et al. (2018), in which fresh mycelium or sporophore was extracted with double distilled H_2_O (ddH_2_O) at 85°C for 6 h. The extracts were centrifuged at 12,000 rpm for 20 min before being concentrated under vacuum. 95% ethanol (EtOH) was added into the supernatant to precipitate the crude polysaccharides. The crude polysaccharides were redissolved in ddH_2_O, and then Sephacryl S-300 gel and DEAE-cellulose column were used to purify polysaccharides. High-performance gel permeation chromatography was employed to detect the molecular weight of the polysaccharides. Briefly, polysaccharides were dissolved in ddH_2_O and filtered through a 0.22-µm membrane filter. The data were analyzed using GPC software (Millennium 32 software; Agilent Technologies, Inc., Santa Clara, CA, USA). For monosaccharide composition analysis, polysaccharides were hydrolyzed with TFA at 110°C for 6 h via acid-catalyzed hydrolysis, and the products were dissolved in ddH_2_O for monosaccharide composition analysis by high-performance liquid chromatography (HPLC) in an evaporative light scattering detector. HPLC was performed under the following conditions: concentration of refined polysaccharides, 20 mg/mL; mobile phase, 75% acetonitrile at 1.4 mL/min; and column oven temperature, 35°C. D-glucose, D-mannose, D-fructose, D-galactose, L-rhamnose, L-arabinose, and D-xylose were used as standard sugars.

Flavonoids were extracted, purified, and identified as described by Shi et al. (2016; 2019), in which 100 g fresh mycelium or sporophore was extracted thrice (4, 2, and 1 h) with 1,000 mL (10 times volume) of 40% EtOH, and then filtered. The filtrate was evaporated under reduced pressure by using a rotary evaporator to obtain the crude extract solution. The crude extract solution was loaded in glass columns which were wet-packed with HPD722 macroporous resin and washed with ddH_2_O, and eluted with 70% EtOH. The eluting solution was concentrated in the rotary evaporation apparatus and vacuum-dried for qualitative and quantitative analysis of total flavonoids using LC-MS/MS(Agilent 1100- triple quadrupole mass spectrometry API4000 with Agilent Poroshell 120 EC-C18 2.7 μm (3 × 50 mm)and Double-flavone of Cortex Platycladi and big posein as internal standard).

### 2.4 Antioxidant and hydroxyl radical scavenging capacity assay

The hydroxyl radical scavenging capacity assay of polysaccharides and flavonoid was carried out by using the hydroxyl free radical scavenging capacity assay kit according to the principle that H_2_O_2_/Fe^2+^ can generate hydroxyl free radical through Fenton reaction (Lyngsie et al., 2018), and the experiment was carried out according to the instructions described in the kit manual (kit BC1325-100T/96S). The total antioxidant capacity of polysaccharides and flavonoid was determined using the total antioxidant capacity assay kit (kit BC1310-50T/48S) based on the reduction of Cu^2+^ to Cu^+^ (Madariaga et al., 2021), and the experiment was carried out according to the instructions described in the kit manual.

### 2.5 Cell proliferation and cytotoxicity assays

The effect of mycelium polysaccharide (JDPS), sporophore polysaccharide (ZDPS), mycelium flavonoids (JHFA), sporophore flavonoids (ZHFA) and zunyimycin C (Wang et al., 2022) on the proliferation rate of immune cells was analyzed as described by Li et al. (2020), in which RAW264.7, Raji and Jurkat cells were inoculated to 96-well plates and incubated with 5% CO_2_ at 37°C. The polysaccharides were dissolved in LPS complete medium at a concentration of 1,000 mg/mL and diluted to final concentrations of 400, 200, 100, 50, 25, 12.5, 6.25, and 3.125 μg/mL. Flavonoids were dissolved in DMSO at a concentration of 1,600 μg/mL and diluted to final concentrations of 100, 50, 25, 12.5, 6.25, and 3.125 μg/mL. LPS was diluted to final concentrations of 5 μg/mL with complete medium as control. After the cells were incubated for 24 h, 100 µL of different concentrations of the drug were added, and RAW264.7 cells were incubated for 48 h, while the Raji and Jurkat cells were incubated for 72 h. Then, the OD450 absorbance values of each cell were detected, and the cell proliferation rate was calculated.

### 2.6 Macrophage phagocytosis assay

Macrophage phagocytosis assay was carried out as described by Djaldetti and Bessler, (2015), in which 4000 RAW264.7 cells were plated and inoculated with 5% CO_2_ at 37°C for 48 h. After discarding the culture medium, 0.4%, 0.04%, 0.01%, and 0.004% trypan blue solutions were added, and the sample was incubated at 37°C for 15 min. The trypan blue solution was discarded, and 200 µL of PBS was added. The sample was rinsed thrice, PBS was discarded, and 100 µL of cell lysate was added to each well to lyse the samples for 2 h. The absorption values were detected at 540 nm, and phagocytosis was calculated.

### 2.7 Measurement of TNF-α, IL-2, IFN-γ, and IL-6

After Jurkat cells and RAW264.7 were inoculated at 5% CO_2_ and 37°C for 24 h, they were incubated for 72 h after the drug was added. The supernatant of cultured cells was collected and centrifuged at 1,000 rpm for 10 min at 4°C. TNF-α, IL-2, IFN-γand IL-6 levels were determined by ELISA. The following ELISAs were employed for the measurement of each parameter: mouse TNF-α (kit SEKM-0034-96T), mouse IL-2 (kit SEKM-0004-96T), mouse IL-6 (kit SEKM-0007-96T), and mouse IFN-γ(kit SEKM-0031-96T) (Avci et al., 2014).

### 2.8 Interventions for immunocompromised mouse model

Thirty-five mice were randomly divided into three groups, namely, the blank control (BLK) group, model group (Cyclophosphamide, CTX), ZDPS low-dose group (ZDPS50, 50 mg/kg/day), ZDPS high-dose group (ZDPS200, 200 mg/kg/day), ZHFA low-dose group (ZHFA50, 50 mg/kg/day), ZHFA high-dose group (ZHFA200, 200 mg/kg/day), and Zunyimycin C group (4.8 mg/kg/day). The mice were intraperitoneally injected with CTX (100 mg/kg) once a day for 3 days to build an immunosuppressive mouse model (Kong et al., 2005; Wang et al., 2022), while the mice in the BLK group were intraperitoneally injected with normal saline (NS) as control. From the fourth day, mice were given normal saline by gavage in the BLK and CTX groups, and other mice were administered with the ZDPS50, ZDPS200, ZHFA50, ZHFA200 and Zunyimycin C for 15 days respectively. Blood samples were obtained from the mice in each group from the orbit, and hemolysin, IL-2, IL-6, IFN-γ, and TNF-α were quantified using ELISA kits as per the manufacturer’s instruction.

On the fourth day, all mice were sensitized by intraperitoneal injection of 0.2 mL of 6% chicken red blood cells, and mice blood samples were collected from the orbit. After resting at room temperature for 1 h, blood samples were centrifuged at 2,000 rpm for 20 min at 4°C, and the supernatant was collected and incubated for 1 h at 37°C with 250 µL of 6% (*v*/*v*) chicken red blood cells and 250 µL of 10% guinea pig serum. The reaction was stopped at 15 min in ice, and the sample was centrifuged at 2,000 rpm for 20 min at 4°C. Approximately 200 µL of supernatant was collected in 96-well plate, and the OD value was measured at 540 nm.

### 2.9 SCFA assay

The feces were collected with sterile centrifuge tubes and stored at −80°C. Fecal SCFA was determined by Shanghai LuMing Biotechnology Company via LC-MS-MS. SCIEX OS-MQ software (Sciex, USA) was employed to identify and integrate MRM transitions with default parameters (Liebisch et al., 2019). The abscissa of the extracted ion current (XIC) diagram of each substance was the retention time (RT) of metabolite detection, and the ordinate was the ion strength (count per second, cps) of ion detection, generating the map of each metabolite. The peak area of the metabolite was interpolated into the regression equation fitted by the standard curve to obtain the concentration of the metabolite. On the basis of the parameters, such as sampling and/or dilution ratio, the concentration of metabolites was calculated, and the absolute content of each metabolite in the actual sample was obtained.

### 2.10 Intestinal flora assay

Murine intestinal microbiota 16S rRNA amplicon sequencing and analysis were conducted by OE Biotech Co., Ltd. (Shanghai, China) (Hu et al., 2022). Total genomic DNA was extracted using a DNA extraction kit following the manufacturer’s instruction. The quality and quantity of DNA were verified with NanoDrop and agarose gel. Extracted DNA was diluted to a concentration of 1 ng/μL and stored at 20°C until further processing. The diluted DNA was used as a template for the PCR amplification of bacterial 16S rRNA genes with the barcoded primers and Takara Ex Taq (Takara). For bacterial diversity analysis, the V3-V4 variable regions of 16S rRNA genes were amplified with universal primers 343F (TACGGRAGGCAGCAG) and 798R (AGGGTATCTAATCCT). The amplicon quality was visualized by gel electrophoresis, purified with AMPure XP beads (Agencourt), and amplified for another round of PCR. After purifying with the AMPure XP beads again, the final amplicon was quantified using the Qubit dsDNA assay kit. Equal amounts of purified amplicon were pooled for subsequent sequencing. Raw sequencing data were stored in FASTQ format. Paired-end reads were then preprocessed using cutadapt software to detect and cut off the adapter. After trimming, paired-end reads were filtered for low-quality sequences, denoised, merged, and detected. The chimaera reads were cut off by using DADA2 with the default parameters of QIIME2 (2020.11). Finally, the software produced the representative reads and ASV abundance table. The representative read of each ASV was selected using the QIIME2 package. All representative reads were annotated and blasted against the Silva database Version 138 (or Unite; 16s rDNA) by using q2-feature-classifier with the default parameters.

### 2.11 Statistical analysis

The data of each group were expressed as the mean ± standard deviation. Statistical analysis was performed using GraphPad Prism 8 software. One-way ANOVA was used for comparison between multiple groups, and t-test was used for comparison between two groups. Statistical significance was considered at *p* < 0.05 (Wang et al., 2022).

## 3 Results

### 3.1 Flavonoids and polysaccharides in *Phellinus igniarius* YASH1

Sequencing results of the PCR products of ITS were BLAST at the NCBI database, and the results showed that the nucleotide sequence similarity between the experimentally collected fungi and the wild *P. igniarius* NW518 in the database, which originated from the Loess Plateau in northern China, was 100%. Therefore, the isolate from this experiment was named *P. igniarius* YASH1.

LC-MS-MS was used to identify and quantify the composition of the total flavonoids from *P. igniarius* YASH1, and the result showed that the flavonoids in ZHFA from wild-type sporophore and laboratory-grown mycelium differed in terms of composition and content (Table 1). In comparison with the mycelium, flavonoids from the sporophore contain unique ingredients such as ihydroquercetin-7-0-rhamnoside, procyanidin B1, anthocyanin B2, p-coumaric acid 1, dglycyrrhizin 1, and cyanidin 2 and lack some flavonoids such as quercetin 1, 2,4,6-tribromophenol 2, vanillin 2 and trans-resveratrol 2. Licorice 1 (581 ng/mL), the largest flavonoid in the sporophore, was not detected in the total flavonoids of the mycelia cultured in the laboratory. By contrast, quercetin 1 (143 ng/mL), which has the highest content in the mycelia cultured in the laboratory, was not detected in the total flavonoids of the wild sporophore.

**TABLE 1:** Flavonoids from *P. igniarius* YASH1.

Compound	ZHFA (ng/mL)	JHFA (ng/mL)	m/z	Retention time RT (min)
Caffeic acid 1	38.3	103	179	3.61
Caffeic acid 3	23.5	76.4	179	3.64
Protocatechuadhyde 1	147	15.4	136	1.94
Proanthocyanidin B1	47.2	0	577	2.23
Proanthocyanidin B2	24.7	0	577	4.52
p-Coumaric acid 1	73	0	163	5.38
p-Coumaric acid 2	130	122	147	7.4
Protocatechuic acid 2	124	98.1	153	1,13
Dihydroquercetin-7-0-rhamnoside	161	0	305	6.69
Ethylvanillin 2	102	90.5	165	7.16
Quercetin 1	0	143	301	9.92
Apigenin 1	30.7	34.7	269	10.8
Apigenin	5.66	2.49	269	10.9
Myricetin 1	93.9	95	317	10.7
Geranisin 1	31.4	29.4	298	11.1
Glycyrrhizic acid 1	4.53	0	821	12.0
2,4,6-Tribromophenol 2	0	108	330	13.5
Licorice 1	581	0	255	17.7
Vanillin 1	0.381	14.2	169	3.3
Vanillin 2	0	8.2	169	3.29
Phenylalanine 1	64.4	47.4	165	0.96
Resveratrol glycoside 1	4.15	24.0	391	6.69
Resveratrol glycoside 2	35.8	37.6	391	6.69
Trans-Resveratrol 2	0	13.9	229	8.65
Cyanidin 2	243	0	286	11

The molecular weights of polysaccharides from *P. igniarius* YASH1 were analyzed by high-performance gel permeation chromatography, and the data (Table 2) show that the number-averaged molecular weight (Mn) of ZDPS is 7,472 Da, the weight-averaged molecular weight (Mp) is 19,319 Da, the molecular weight (Mw) is 20,323, Z average molecular weight (Mz) is 40,689, and the molecular weight z+1-average (Mz+1) is 64,771 with corresponding values in JDPS of 9,795, 23,457, 21,270, 43,172, and 65,541, respectively.

**TABLE 2:** Molecular weight of *P. igniarius* YASH1 polysaccharides.

Polysaccharides	M_n_/Da	M_w_/Da	M_p_/Da	M_z_/Da	M_z_+1/Da
ZDPS	7,472	20,323	19,319	40,689	64,771
JDPS	9,795	23,457	21,270	43,172	65,541

### 3.2 Hydroxyl radical scavenging and antioxidant capacity activity of polysaccharides and flavonoids

**FIGURE 1:**
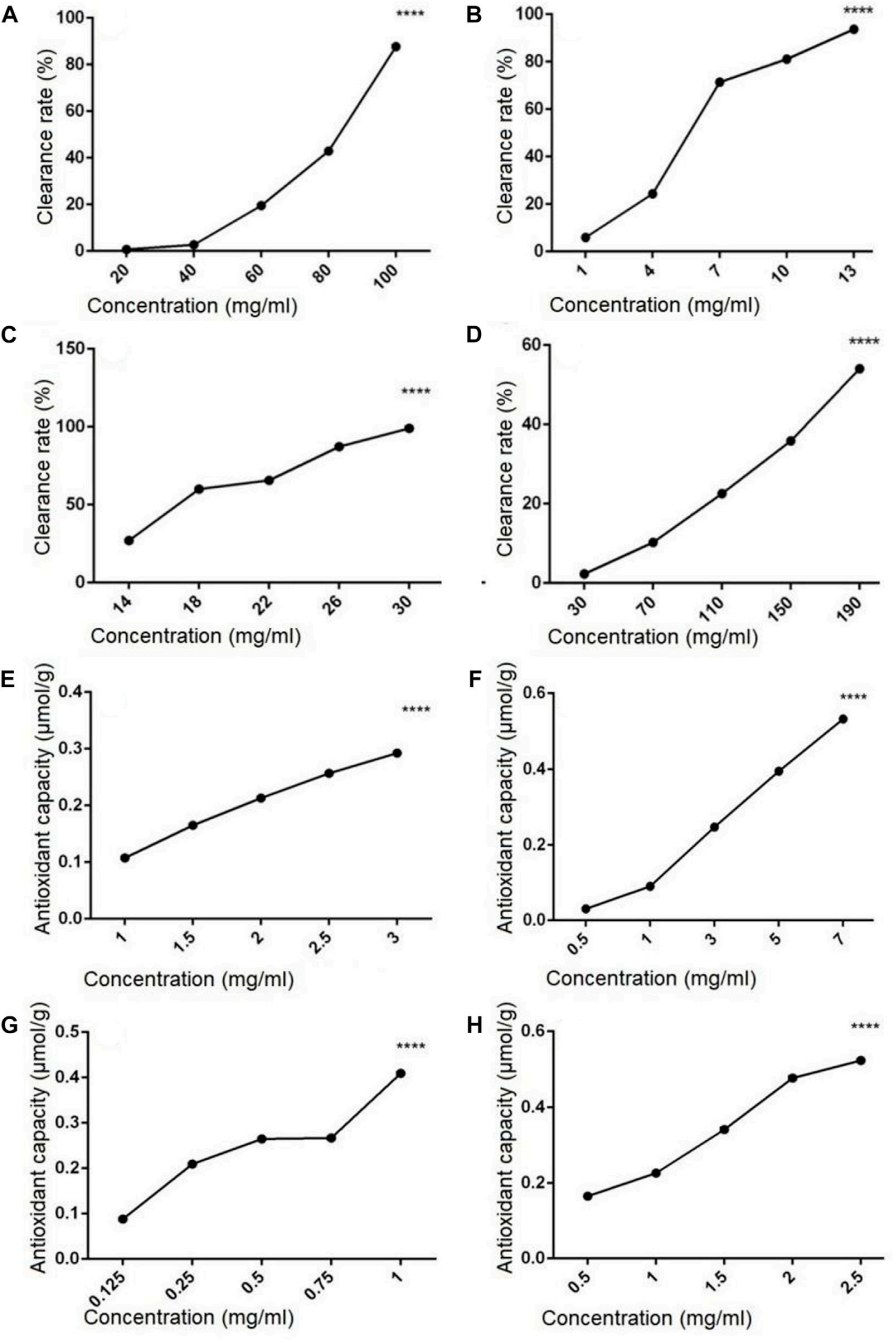
Hydroxyl radical scavenging and antioxidant capacity of polysaccharides and flavonoids **(A–D)** Hydroxyl radical scavenging capacity of polysaccharides and flavonoids. **(E–H)** Antioxidant capacity of flavonoids and polysaccharides. **(A)** ZDPS. **(B)** JDPS. **(C) **ZHFA. **(D)** J HFA. **(E)** ZDPS. **(F)** JDPS. **(G)** ZHFA. **(H)** JHFA. ****: statistically significant (*p* < 0.0001).

The hydroxyl radical scavenging activity of polysaccharides and flavonoids extracted from sporophore or laboratory-grown mycelium of *P. igniarius* YASH1 was analyzed, and the result is shown in Figure 1. Results showed that the hydroxyl radical scavenging rate of flavonoids and polysaccharides increased with the increasing in concentration at a certain concentration range, indicating a concentration-dependent effect (Figures 1A–D). In the concentration range of 20–100 mg/mL, the hydroxyl radical scavenging rate of ZDPS (Figure 1A) increased with the increasing in concentration, which was concentration-dependent and statistically significant (*p* < 0.0001), while JDPS (Figure 1B) showed a concentration-dependent activity of hydroxyl radical scavenging rate in the range of 1–13 mg/mL (*p* < 0.0001). For flavonoids, in the concentration range of 14–30 mg/mL, the hydroxyl radical scavenging rate of ZHFA (Figure 1C) increased with the increasing in concentration, which was concentration-dependent and statistically significant (*p* < 0.0001), while JDFA (Figure 1D) showed a concentration-dependent activity of hydroxyl radical scavenging rate in the range of 30–190 mg/mL (*p* < 0.0001).

The antioxidant capacity of polysaccharides and flavonoids from the sporophore or laboratory-grown mycelium of *P. igniarius* YASH1 was detected. The results are shown in Figure 1, in which the ZDPS and JDPS of polysaccharides and the ZHFA and JHFA of flavonoids have antioxidant capacity (Figures 1E–H). The ZDPS (Figure 1E) and JDPS (Figure 1F) of polysaccharides were in the concentration ranges of 1–3 and 0.5–7 mg/mL, respectively, and the total antioxidant increased with the increasing in concentration. A certain significant concentration dependence (*p* < 0.0001) was observed. Both the ZHFA (Figure 1G) and JHFA (Figure 1H) flavonoids were in the concentration ranges of 0.125–1 and 0.5–2.5 mg/mL, respectively. The total antioxidant increased with the increasing in concentration, and a significant concentration dependence (*p* < 0.0001) was observed.

### 3.3 Effect of polysaccharides and flavonoids on cell proliferation

**FIGURE 2:**
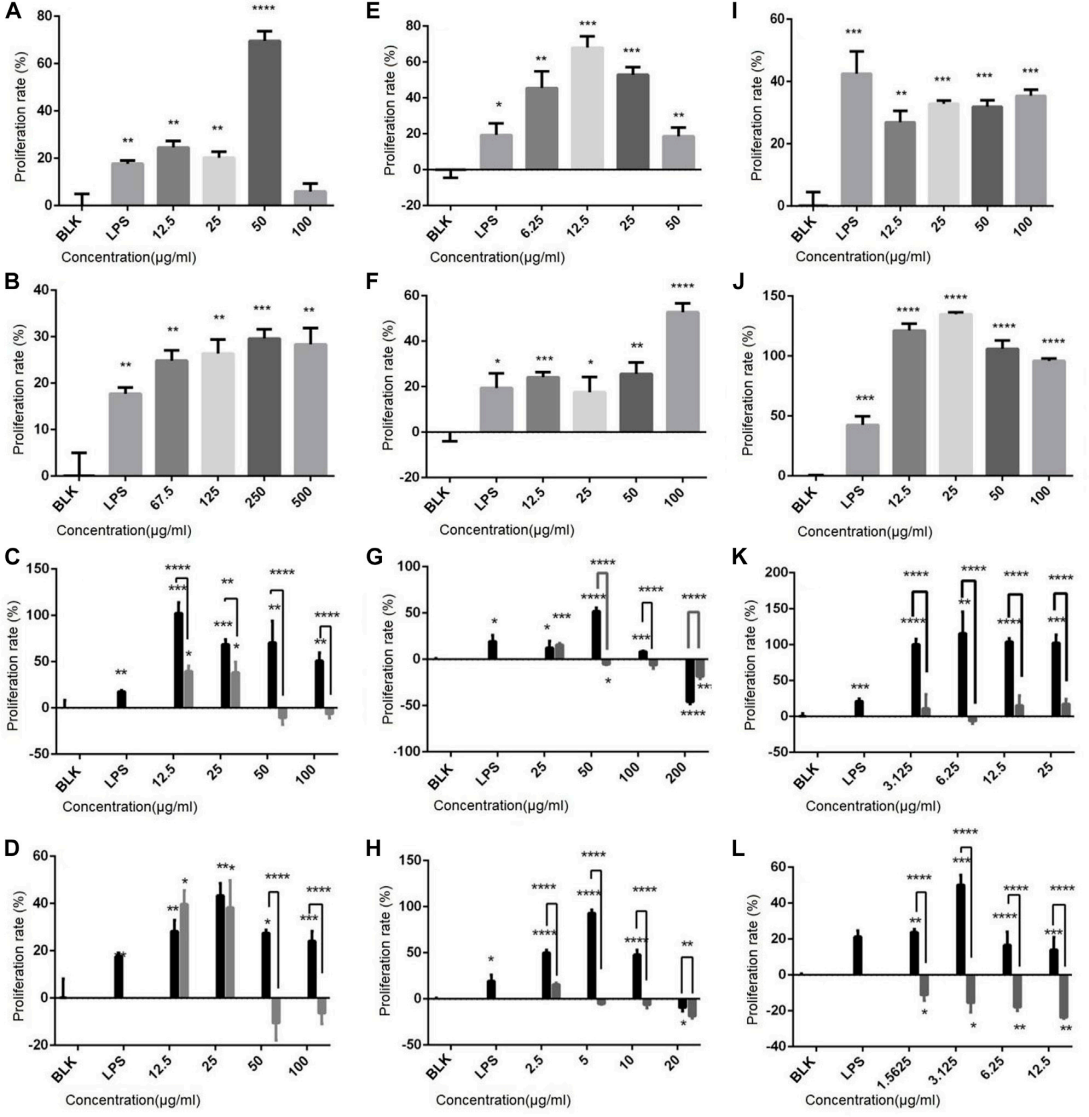
Effect of polysaccharides and flavonoids on cell proliferation **(A–D)** Effects of ZDPS, JDPS, ZHFA and JHFA on the proliferation capacity of T lymphocytes Jurkat cells. **(E–H)** Effects of ZDPS, JDPS, ZHFA and JHFA on the proliferation capacity of B lymphocytes Raji cells. **(I–L)** Effects of ZDPS, JDPS, ZHFA and JHFA on the proliferation capacity of macrophage Raw264.7 cells. BLK: Blank control. LPS: lipopolysaccharide. *: significant difference (*p* < 0.05), **: significant difference (*p* < 0.01), ***: significant difference (*p* < 0.001), ****: significant difference (*p* < 0.0001).

The effect of drugs on the proliferation rate of immune cells was determined, and results show (Figure 2) that both polysaccharides and flavonoids promote the cell proliferation of the three cell lines. For example, the sporophore polysaccharides ZDPS has a proliferative effect on Jurkat cells and showed the strongest stimulating effect (74.44%) at 50 μg/mL (*p* < 0.0001; Figure 2A). It also has a proliferative effect on Raji cells and showed the strongest stimulating effect (62.33%) at 12.5 μg/mL (*p* < 0.0001) (Figure 2E), while in Raw264.7 cells, the strongest stimulating effect (36.57%) was observed at 100 μg/mL (*p* < 0.001; Figure 2I), which was statistically significant compared with the blank control (BLK) group. JDPS, the mycelium polysaccharide, has a proliferative effect on Jurkat cells and showed the strongest stimulating effect (27.5%) at 250 μg/mL (*p* < 0.0001; Figure 2B). It also has a proliferative effect on Raji cells and showed the strongest stimulating effect (53.85%) at 100 μg/mL (*p* < 0.0001; Figure 2F). In Raw264.7 cells, the strongest stimulating effect (136.57%) was observed at 25 μg/mL (*p* < 0.0001; Figure 2J) which is statistically significant compared with the negative control. The sporophore flavonoids ZHFA has a proliferative effect on Jurkat cells, showed the strongest stimulating effect (88.85%) at 12.5 μg/mL (*p* < 0.001; Figure 2C), and had a proliferative effect on Raji cells, showing the strongest stimulating effect (56.14%) at 50 μg/mL (*p* < 0.0001; Figure 2G), while in Raw264.7 cells, the strongest stimulating effect (100.47%) was observed at 6.25 μg/mL (*p* < 0.001; Figure 2K), and the effect was statistically significant compared with the negative control. JHFA, the mycelium flavonoid with a proliferative effect on Jurkat cells and showed a strongest stimulating effect (37.5%) at 25 μg/mL (*p* < 0.005; Figure 2D), and had a proliferative effect on Raji cells that showed the strongest stimulating effect (54.29%) at 5 μg/mL (*p* < 0.0001; Figure 2H). In Raw264.7 cells, the strongest stimulating effect (48.36%) was observed at 3.125 μg/mL (*p* < 0.0001; Figure 2L), and the effect was statistically significant compared with the negative control.

**FIGURE 3:**
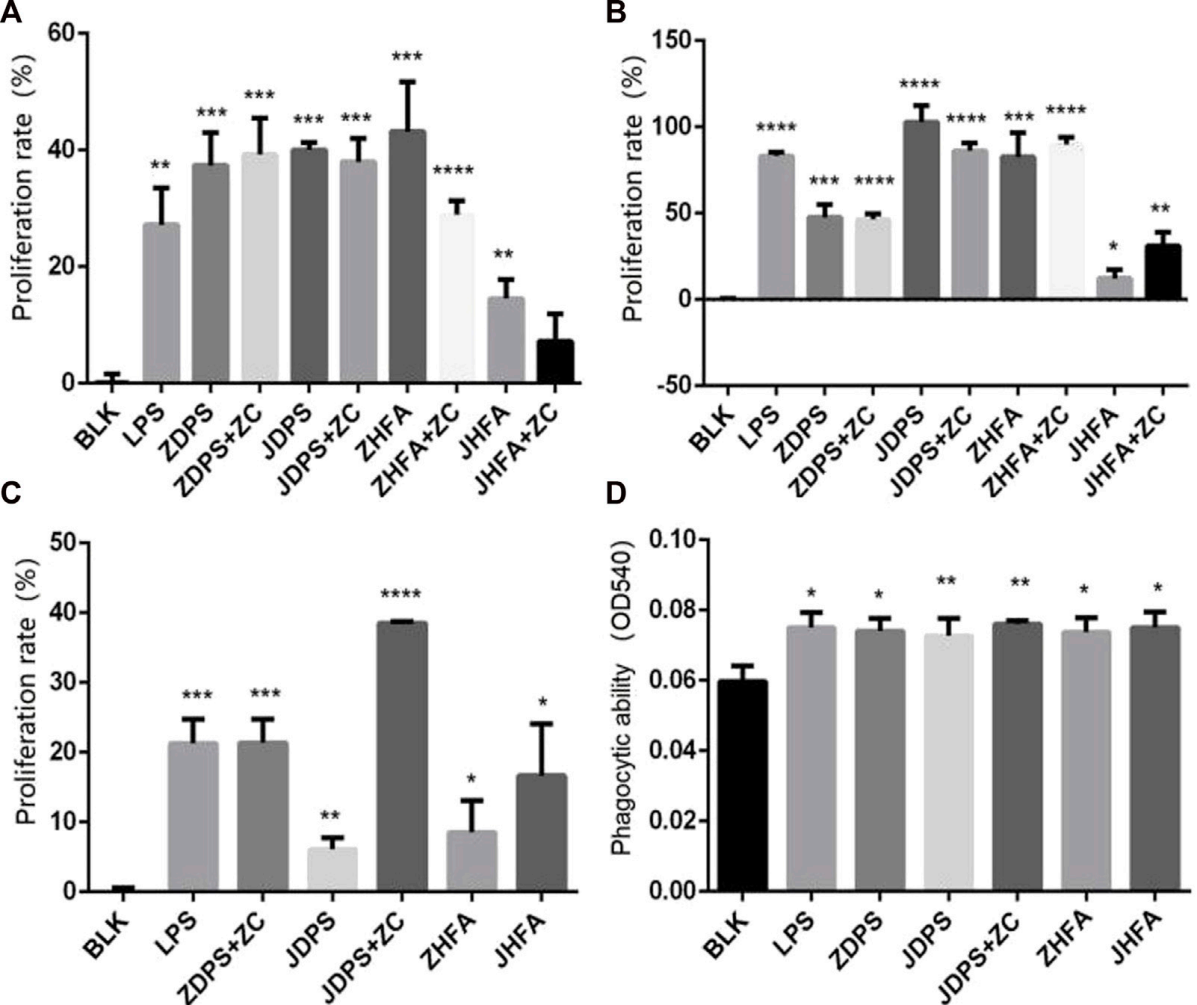
Effects of drugs on cell proliferation and phagocytic ability **(A–C)** Effects of drugs or concomitant drugs on cell proliferation. **(D)** Effects of drugs or concomitant drugs on phagocytosis of RAW264.7. ZC: zunyimycin C. BLK: Blank control. LPS: lipopolysaccharide. *: significant difference (*p* < 0.05); **: significant difference (*p* < 0.01); ***: significant difference (*p* < 0.001); ****: significant difference (*p* < 0.0001).

The effect difference between drug combinations and drugs alone was compared using half the drug concentration of the best effect concentration of ZDPS, JDPS, ZHFA, and JHFA and half the concentration of the best effect concentration of zunyimycin C. The combination of ZDPS (50 μg/mL), JDPS (500 μg/mL), ZHFA (12.5 μg/mL), and JHFA (5 μg/mL) with zunyimycin C (1.25 μg/mL) was used to detect the effect of drug combination on the proliferation of Jurkat cells. The results in Figure 3 show that compared with the BLK group, the drugs ZDPS, ZDPS+Zun C, JDPS, JDPS+Zun C, ZHFA, ZHFA+Zun C, JHFA, and JHFA+Zun C promoted the proliferation of Jurkat cells with a statistical difference (*p* < 0.01). No significant difference was observed between ZDPS and ZDPS+Zun C. Similarly, no significant difference was observed between JDPS and JDPS+Zun C, ZHFA and ZHFA+Zun C, and JHFA and+Zun C (Figure 3A). ZDPS (12.5 μg/mL), JDPS (100 μg/mL), ZHFA (50 μg/mL), and JHFA (50 μg/mL) were used in combination with zunyimycin C (2.5 μg/mL). ZDPS, ZDPS+Zun C, JDPS, JDPS+Zun C, ZHFA, ZHFA+Zun C, JHFA, and JHFA+Zun C promoted Raji cell proliferation compared with the BLK group with a statistical difference (*p* < 0.05). No significant difference was observed between JDPS and ZDPS+Zun C. Similarly, no significant difference was observed between JDPS and JDPS+Zun C, ZHFA and ZHFA+Zun C (Figure 3B). ZDPS (100 μg/mL), JDPS (25 μg/mL), ZHFA (6.25 μg/mL), and JHFA (3.125 μg/mL) were used in combination with zunyimycin C (2.5 μg/mL). ZDPS, JDPS, JDPS+ZC, ZHFA, and JHFA promoted the proliferation of Raw 264.7 cells compared with the BLK group, with a statistical difference (*p* < 0.001), and JDPS+Zun C had a higher cell proliferation rate than JDPS. A statistical difference was observed (*p* < 0.05), but the cells in the drug combination were all dead, except for JDPS+Zun C (Figure 3C).

The effects of polysaccharides and flavonoids on the phagocytic ability of macrophages were determined using the trypan blue method to detect the phagocytic ability of RAW264.7 cell lines according to the change of OD540 after drug use. After stimulating macrophages by using various drugs and drug combinations, the phagocytic ability of macrophages was improved, which was statistically significant compared with the negative control (*p* < 0.05; Figure 3D).

### 3.4 Effects of polysaccharides and flavonoids on cytokine secretion

**FIGURE 4:**
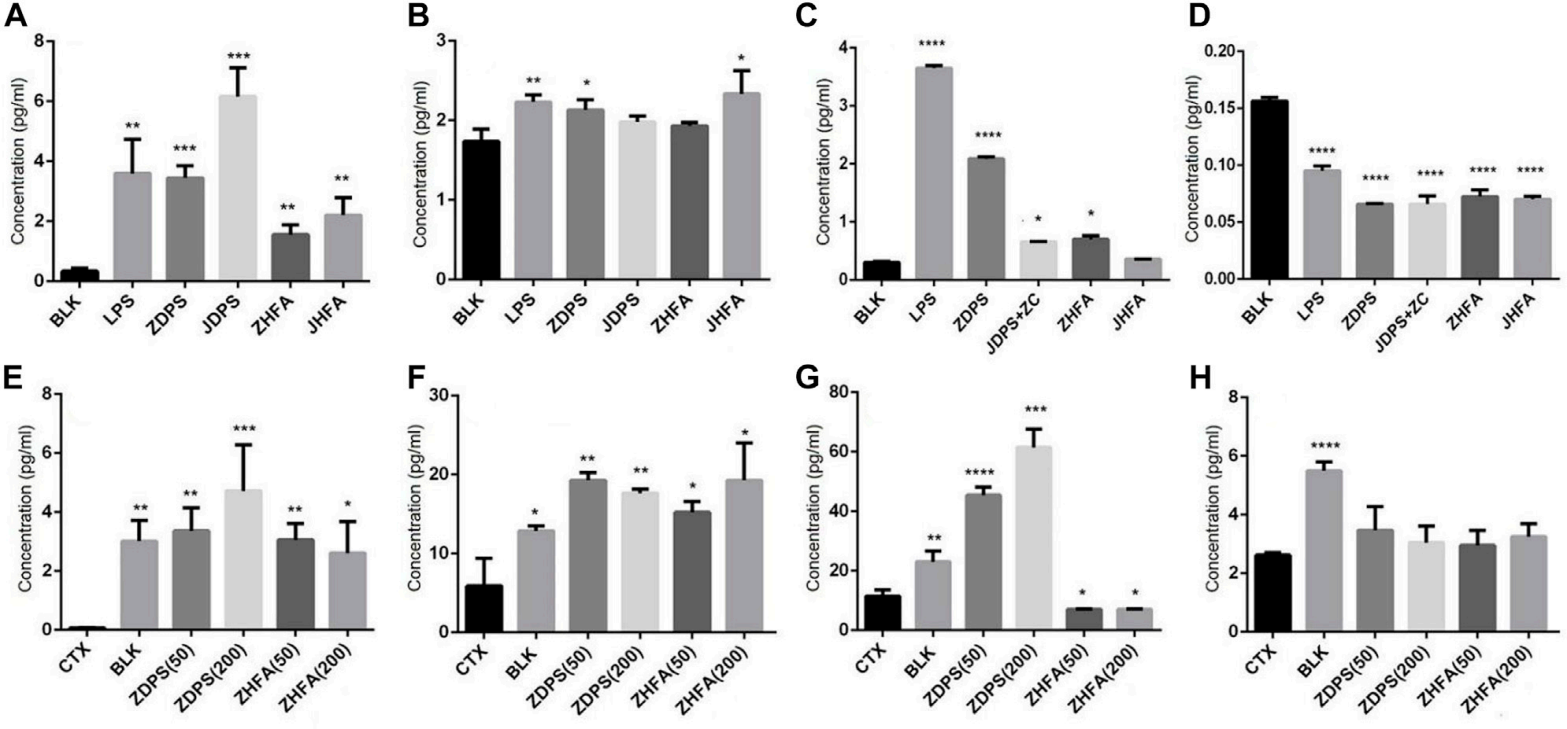
Effects of polysaccharides and flavonoids on cytokine secretion **(A–D)** cytokine secretion*in vitro*. **(E–H)** Cytokine secretion *in vivo*. **(A)** IL-2 secreted by Jurkat cells. **(B)** IFN-γ secreted by Jurkat cells. **(C)** IL-6 secreted by macrophages cell RAW264.7. **(D)** TNF-α secreted by macrophages cell RAW264.7. **(E)** IL-2 secreted in mice. **(F)** IL-6 secreted in mice. **(G)** TNF-α secreted in mice. **(H)** IFN-γ secreted secreted in mice. BLK: Blank control. LPS: lipopolysaccharide. ZC: zunyimyicn C; BLK: Blank control; CTX:Cyclophosphamide; ZDPS50: ZDPS 50 mg/kg/d; ZDPS200: ZDPS 200 mg/kg/d; ZHFA50: ZHFA 50 mg/kg/d; ZHFA200: ZHFA 50 mg/kg/d. *: significant difference (*p* < 0.05); **: significant difference (*p* < 0.01); ***: significant difference (*p* < 0.001); ****: significant difference (*p* < 0.0001).

The levels of cytokines *in vivo* and *in vitro* were measured, and the secretion of IL-2 and IFN-γ of Jurkat cells, as well as IL-6 and TNF-α of RAW264.7 cells were detected with cytokine detection kits. Figure 4 shows that the secretion levels of IL-2 increased from 0.29 pg/mL to 3.5, 3.82, 5.45, 1.56, and 2.36 pg/mL when Jurkat cells were stimulated with LPS, ZDPS, JDPS, ZHFA, and JHFA respectively, and the difference was statistically significant (*p* < 0.01; Figure 4A).

When Jurkat cells were stimulated with LPS, ZDPS, JDPS, ZHFA, and JHFA, the secretion levels of IFN-γ increased from 1.68 pg/mL to 2.28, 2.20, 2.05, 1.9, and 2.2 pg/mL respectively, and the difference was statistically significant (*p* < 0.01; Figure 4C). In RAW264.7 cells, the secretion levels of IL-6 increased from 0.276 pg/mL to 3.63 pg/m, 2.10, 0.65, 1.56, and 0.64 pg/mL when the cells were stimulated with LPS, ZDPS, JDPS, ZHFA, and JHFA, respectively, and the difference was statistically significant (*p* < 0.01; Figure 4B). The secretion levels of TNF-α increased from 0.154 pg/mL to 3.63, 2.10, 0.65, 1.56, and 0.64 pg/mL when the cells were stimulated with LPS, ZDPS, JDPS, ZHFA, and JHFA, respectively, and the difference was statistically significant (*p* < 0.01; Figure 4D). After treatment with polysaccharides or flavonoids, the expression level of cytokines in immunosuppressive animal models was detected using the ELISA kit. Results showed that the level of IL-2 in CTX significantly decreased (*p* < 0.01) and the levels of IL-2 in mice medicated with polysaccharide and flavone increased (*p* < 0.05; Figure 4E) compared with the BLK group. Similarly, the level of IL-6 in the CTX group was significantly lower than that in the BLK group (*p* < 0.05). After treatment with polysaccharides and flavonoids, the level of IL-6 in mice significantly increased compared with that in the CTX group (*p* < 0.05; Figure 4F). The expression level of TNF-α in the CTX group was significantly lower than that in the BLK group (*p* < 0.01), but the effect was different after drug treatment, in which only the level of TNF-α treated with ZDPS significantly increased (*p* < 0.001; Figure 4G). The level of IFN-γ, like IL-2 and IL-6, was significantly lower in the CTX group than in BLK group. The expression level of IFN-γ in mice interfered by polysaccharides or flavonoids increased, but the difference was not significant (Figure 4H).

### 3.5 Effect of drugs on the recovery of organ index in immunocompromised mice

After medication, the mice were sacrificed by necking, and the relevant organs were obtained to calculate the organ index. The effect of the drug on the recovery of the organ index of the immunocompromised mice was analyzed. Supplementary Figure S1 shows the effects of each drug on the organs of mice.

In comparison with the BLK group, the thymus and spleen index were significantly lower in the CTX group (*p* < 0.01), while flavonoids and polysaccharides showed a certain recovery ability to the thymus of CTX-treated mice at different concentrations (Supplementary Figure S1A). Higher concentrations of polysaccharides (*p* < 0.05) and lower concentrations of flavonoids (*p* < 0.01) showed a certain recovery ability to the spleen of CTX-treated mice (Supplementary Figure S1B). However, the liver (Supplementary Figure S1C), heart, lung, and kidney index in the CTX-, BLK- and polysaccharide- or flavonoid-medicated animals had no obvious difference in the final week of dosing.

### 3.6 Effects of drugs on the biosynthesis of SCFAs in immunocompromised mice

**FIGURE 5:**
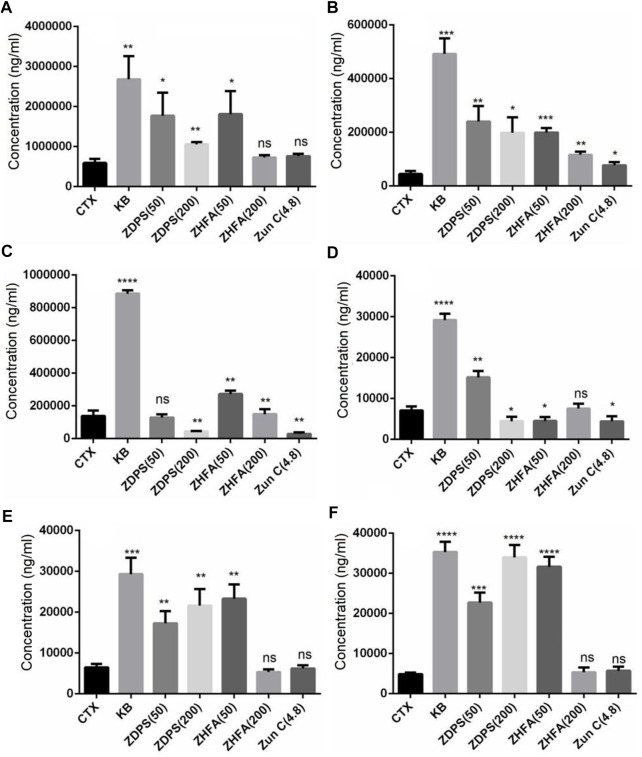
Intestinal SCFAs content after drugs treatment. **(A)** Acetic acid; **(B)** Propionic acid; **(C)** Butyrate; **(D)** Valeric acid; **(E)** Isovaleric acid; **(F)** Isobutyric acid.*: significant difference (*p* < 0.05); **: significant difference (*p* < 0.01); ***: significant difference (*p* < 0.001); ****: significant difference (*p* < 0.0001).

The composition and content of SCFAs were calculated using the UPLC-ESI-MS/MS, and SCIEX OS-MQ software was used to analyze the difference of SCFAs in stools between the seven groups of mice. In comparison with the normal BLK group mice, the intestinal acid, butyrate, caproic acid, and isobutyric acid in the mice treated with CTX drugs and the contents of isovaleric acid, valic acid, and probionic acid decreased significantly by 74.79%, 82.83%, 79.90%, 86.14%, 76.41%, 77.01%, and 90.28% respectively (Table 3). However, compared with the model mice treated with CTX, the changes in SCFA contents in the intestinal tract of mice were not consistent after intervention with different drugs (Figure 5). Both high-dose (*p* < 0.05) and low-dose drug ZDPS (*p* < 0.01) and low-dose ZHFA (*p* < 0.05) can restore the ability of intestinal flora to produce acetic acid to a certain extent, while high-dose ZHFA and antibiotic zunyimycin C have no effect on the recovery of intestinal acetic acid in mice (Figure 5A). For the biosynthesis of propionic acid in the intestine, five drugs all showed positive intervention effects. Therefore, the ability of intestinal flora to produce propionic acid can be restored to a certain extent by using high-dose (*p* < 0.01) and low-dose drugs ZDPS (*p* < 0.05), as well as high-dose (*p* < 0.001) and low-dose ZHFA (*p* < 0.01). The antibiotic zunyimycin C also promoted the recovery of propionic acid production in the intestine of mice (*p* < 0.01; Figure 5B). For butyric acid, the effects of these five drugs are very different, of which high dose (*p* < 0.01) and low dose of ZHFA (*p* < 0.01) can promote the intestinal flora to produce butyric acid to a certain extent, while low dose of ZDPS did not affect the recovery of butyric acid in the intestinal tract of mice. However, high-dose ZDPS had a negative effect on butyric acid production in the intestinal tract of mice similar to zunyimycin (*p* < 0.01; Figure 5C). For valeric acid biosynthesis, except for the positive effect of low-dose ZDPS on the recovery of intestinal valeric acid production in mice (*p* < 0.01), high-dose ZHFA had no effect on the recovery of intestinal valeric acid production in mice. However, high dose of ZDPS, low dose of ZHFA, and zunyimycin had a negative effect on the production of valeric acid in the intestine of mice (*p* < 0.01; Figure 5D). For isovaleric acid, high dose of ZHFA and zunyimycin had no effect on the production of isovaleric acid in mouse intestine, whereas high (*p* < 0.01) and low dose of ZDPS (*p* < 0.01) and low dose of ZHFA (*p* < 0.01) promoted the production of isovaleric acid in mouse intestine (Figure 5E). The effect of the drug on the production of isobutyric acid is very similar to that of isovaleric acid, except that high doses of ZHFA and zunyimycin have no effect on the production of isobutyric acid in the intestine of mice, whereas high (*p* < 0.0001) and low doses of ZDPS (*p* < 0.001) and low doses of ZHFA (*p* < 0.0001) promote the production of isobutyric acid in the intestine of mice (Figure 5F). Notably, all drugs inhibited the production of caproic acid in the intestine of CTX model mice. Consequently, the presence of caproic acid in the sample could not be detected.

**TABLE 3:** SCFAs in intestinal tract of mice.

		Metabolite concentration in sample (ng/g)						
ID	Metabolites	BLK	CTX	ZDPS50	ZDPS200	ZHFA50	ZHFA200	ZUN C
1	Acetate	2,349,158.9	592,236.3	1,436,809.4	1,025,399.0	1,478,241.0	694,809.05	728,426.4
2	Butyrate	886,153.1	152,134.6	128,885.0	43,546.6	272,639.3	149,775.65	25,477.3
3	Caproate	1,063.6	213.8	0	0	0	0	0
4	Isobutyrate	35,617.9	4,936.5	22,371.1	36,646.0	31,972.1	4,687.5	5,732.2
5	Isovalerate	29,310.1	6,913.7	17,279.7	20,956.7	23,652.7	5,571.8	6,339.1
6	Valerate	30,851.4	7,092.0	14,853.6	4,615.9	3,878.3	7,601.1	4,455.7
7	Propionate	458,855.6	44,607.2	273,512.4	231,513.6	209,024.5	122,882.3	83,800.0

### 3.7 Drug treatment modified gut microbiota in immunocompromised mice

**FIGURE 6:**
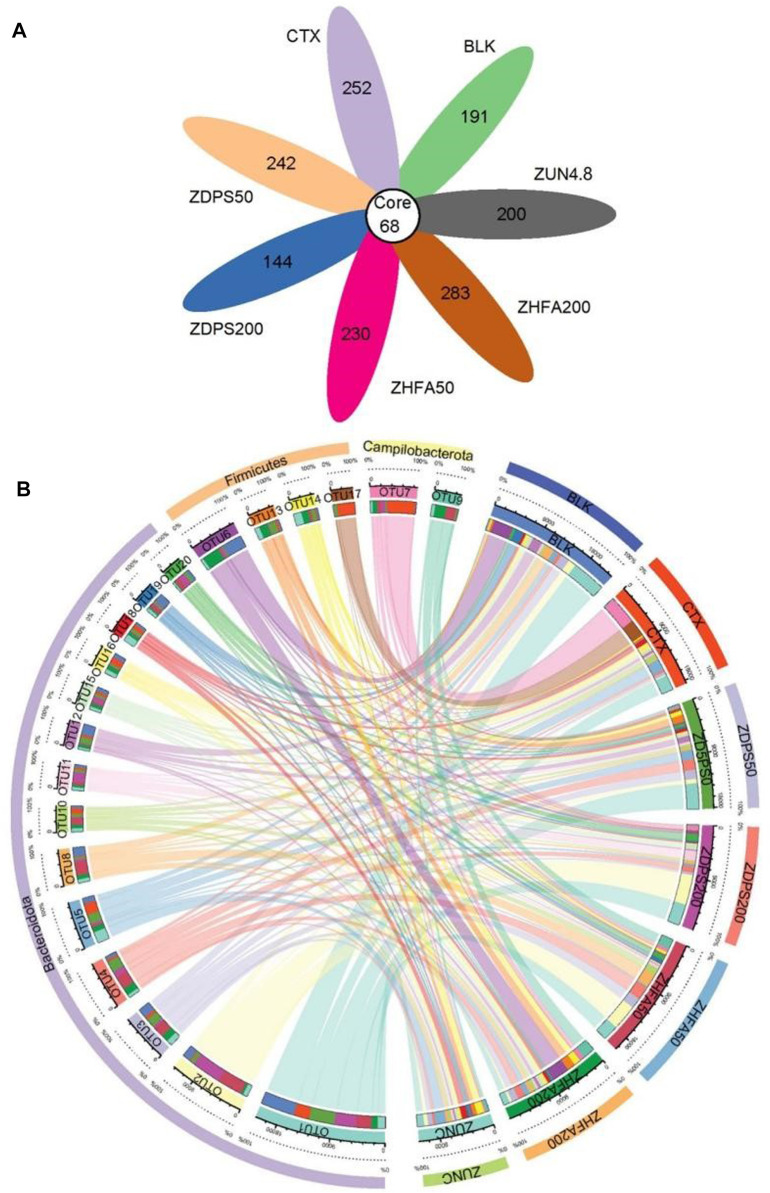
ASVs difference and Collinearity in immunocompromised mice **(A)** The number in the core represents the ASVs common to all samples. The number on the petal represents the total ASVs minus the number of ASVs common to all samples. **(B)** Relationship between circos sample and species of top 20 OTU. First circle: phylum level classification of each sample; Second circle: percentage information of ASV relative abundance; Third circle: ASV and the sample main block, which are distinguished by different colors and labels, the scale around the block is the absolute abundance information of ASV; Fourth circle: ASV and sample sub blocks, corresponding to the main block (the third circle), showing the abundance in each sample and the abundance information of each ASV contained in each sample; Fifth circle: corresponds to ASV and sample sub block (the fourth circle), and the connection shows the associated information of ASV and sample. ZUN C: zunyimyicn C; BLK: Blank control; CTX:Cyclophosphamide; ZDPS50: ZDPS 50 mg/kg/d; ZDPS200: ZDPS 200 mg/kg/d; ZHFA50: ZHFA 50 mg/kg/d; ZHFA200: ZHFA 50 mg/kg/d. OTU1: Rikenellaceae; OTU2: *Bacteroides*; OTU3: Muribaculaceae; OTU4: Alloprevotella; OTU5: Muribaculaceae; OTU6: *Lactobacillus*; OTU7: *Helicobacter*; OTU8: Muribaculaceae; OTU9: *Helicobacter*; OTU10: Muribaculaceae; OTU11: Muribaculaceae; OTU12: Muribaculaceae; OTU13: xylanophIlum; OTU14: *Lactobacillus*; OTU15: Alistipes; OTU16: Odoribacer; OTU17: Lachnospiracfae; OTU18: Muribaculaceae; OTU19: Prevotellaceae; OTU20: Muribaculaceae.

The effects of drug treatment on intestinal flora in immunocompromised mice were investigated by analyzing the 16S rDNA sequencing of the stools. The 16S rDNA gene amplicon sequencing method (V3–V4 region) was used, and 78,078–80,736 raw reads and 35,564–52,143 clean tags were generated for each sample, with clean tags that include 706 ASVs ranging from 212 to 351 for different drug treatments. Each group had specific ASVs (Figure 6A).

**FIGURE 7:**
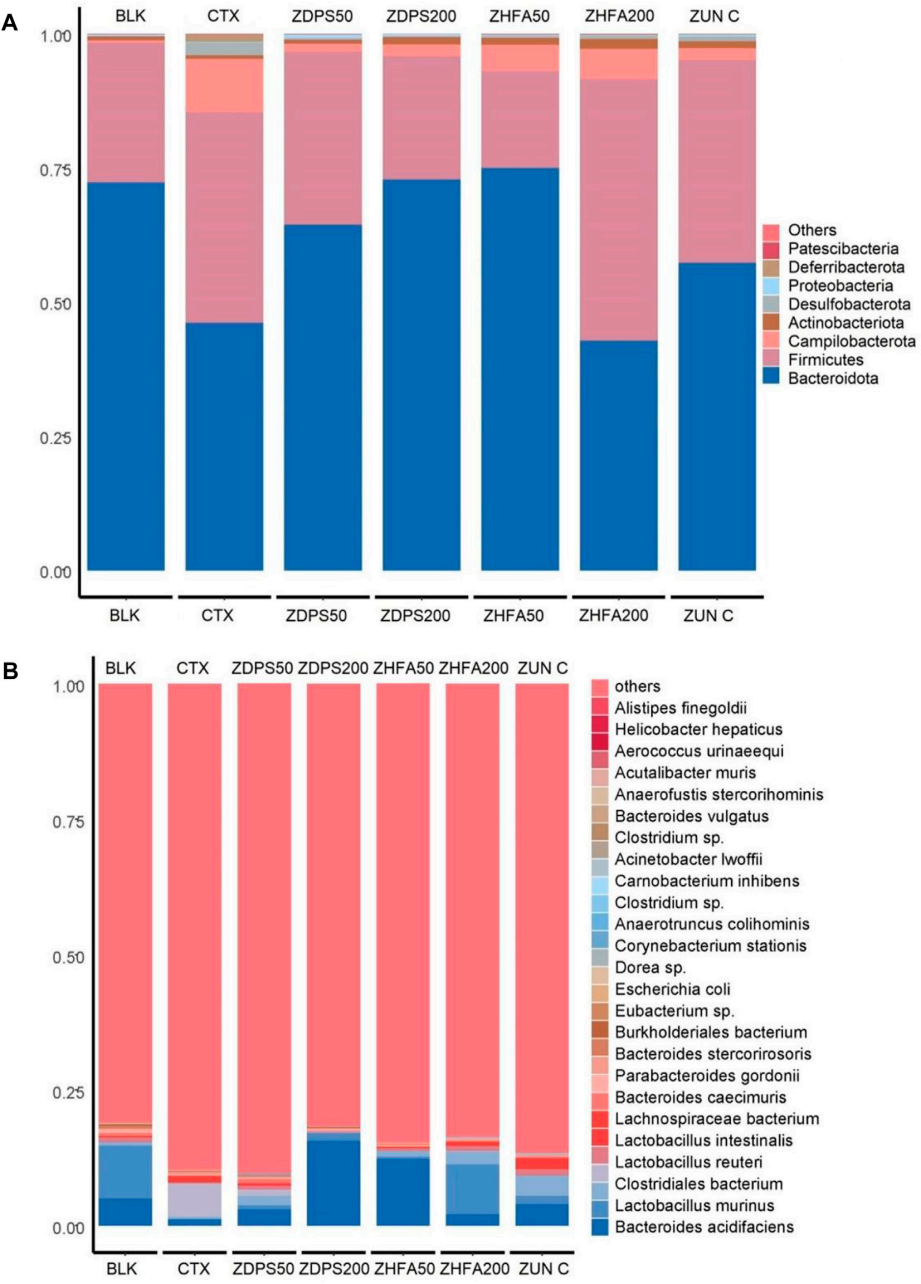
Relative abundance of bacteria in immunocompromised mice. **(A)** Relative abundance of the top 30 bacteria at the phylum level. **(B)** Relative abundance of the top 30 bacteria at the species level. The figure shows the proportion of species in all samples without grouping. The abscissa is the sample analysis name, and the ordinate is the proportion of different species in the sample. Different species are distinguished by different colors, and the annotation information of species is on the right. ZUN C: zunyimyicn C; BLK: Blank control; CTX: Cyclophosphamide; ZDPS50: ZDPS 50 mg/kg/d; ZDPS200: ZDPS 200 mg/kg/d; ZHFA50: ZHFA 50 mg/kg/d; ZHFA200: ZHFA 50 mg/kg/d.

The seven groups of experimental animals shared 68 ASVs (Core ASVs: represents the ASVs common to all samples). Among the 68 common core ASVs, the largest is *Muribaculateae* with 32 ASVs, followed by *Rikenellacaee* with 8 ASVs (including 6 *Alistipes*), *Lachnospiraceae*, and *Bacteroids* with 4 ASVs. *Prevotellelaceae*, *Oscillospiraceae*, and *Enterorhabdus* have three ASVs, while *Lactobacillus* and *Helicobacter* have two ASVs. *Negatinibacillus*, *Biophila*, *Odoribactor*, *Ruminococcus*, *Colorectobacter*, *Alloprevotella*, *Escherichia coli*, and *Escherichia Shigella* have only one ASV. The ASVs among the groups were significantly different. For example, the CTX group has 320 ASVs, the BLK group has 259 ASVs, and Zunyimycin C, ZDPS200, ZDPS50, ZHFA200, and ZHFA50 have 268, 212, 310, 351, and 298ASVs, respectively. The relationship between circle samples and specifications was analyzed, and the data are shown in Figure 7B. Results show that among the 20 OTUs with the highest abundance (sorted from high to low and numbered according to abundance), Bacteroidote has the highest number of OTUs, including 15 OTUs (OTU1-5, OTU8, OTU10-12, OTU15-16, and OTU18-20), followed by firms, including OTU6, OTU13-14, and OTU17. Finally, Campilobacterota includes OTU7 and OTU9. OTU1 was used as an example. In comparison with the normal group of animals, the abundance of OTU1 in mice decreased by about 2/3 after using CTX. The drugs ZDPS50 and ZDPS200 can promote its proliferation and restore its abundance, but ZHFA50 had no effect, while the use of drugs ZHFA200 and zunyimycin resulted in the continued decline of OTU1 in the animal intestine. For OTU2, after the use of drug CTX, the animal OTU2 abundance decreased, and the drugs ZDPS50, ZHFA200, and zunyimycin could not restore their abundance, whereas ZDPS200 and ZHFA50 could significantly increase their abundance. For OTU3, ZDPS50 and ZHFA50 can improve its abundance, but other drugs cannot change the abundance of OTU3 in CTX-treated animals. OTU4 is different. CTX can reduce its abundance to some extent, but zunyimycin has no effect, whereas the other four drugs significantly increase its abundance. CTX can increase the abundance of OTU5. In addition to being irreversible by ZDPS50, the four other drugs can restore the abundance of OTU5 to near or even below the normal level to a certain extent. CTX can reduce the abundance of OTU8. This condition can be reversed by ZHFA50, and the four other drugs may not affect the recovery of the abundance of OTU8. ZDPS200 can aggravate the decrease in OTU10 abundance caused by CTX, but the four other drugs can increase its abundance. OTU11 was slightly affected by CTX. ZDPS200 can increase its abundance, whereas the other drugs only slightly affected it. Except CTX can reduce the abundance of OTU12 and ZDPS50 can increase its abundance, other drugs only have a slight impact. For OTU15, all treatments only slightly affect its abundance. Zunyimycin and ZHFA200 had little effect on the OTU16 abundance of mice treated with CTX, while ZDPS50, ZDPS200, and ZHFA50 could promote their recovery. ZHFA200 and ZDPS50 slightly affected the OTU18 abundance of mice treated with CTX, while ZDPS200 and ZHFA50 can promote their recovery, and zunyimycin can further increase the OTU18 abundance. All drugs can promote the reduction of the abundance of OTU19 and OTU20 caused by the drug CTX to return to normal. For bacteria such as OTU6, OTU13-14, and OTU17 belonging to Firmicutes, a different finding was obtained. CTX significantly reduced the abundance of OTU6. In addition to zunyimycin, other drugs cannot restore the abundance. CTX only slightly affected OTU13, which has a low abundance, but ZDPS200 and ZHFA50 had no effect, whereas the three other drugs can increase its abundance. Both drugs can increase the abundance of OTU14 with decreased CTX, and decrease the abundance of OTU17 with increased CTX. For OTU7 and OTU9 bacteria belonging to Campilobacterota, drugs can significantly reduce the abundance of OTU7, which has been greatly increased by CTX, while ZDPS50 and ZDPS200 have little effect on CTX, but the three other drugs can increase the abundance of OTU9.

### 3.8 Drug treatment modified the abundance of bacteria in immunocompromised mice

**FIGURE 8:**
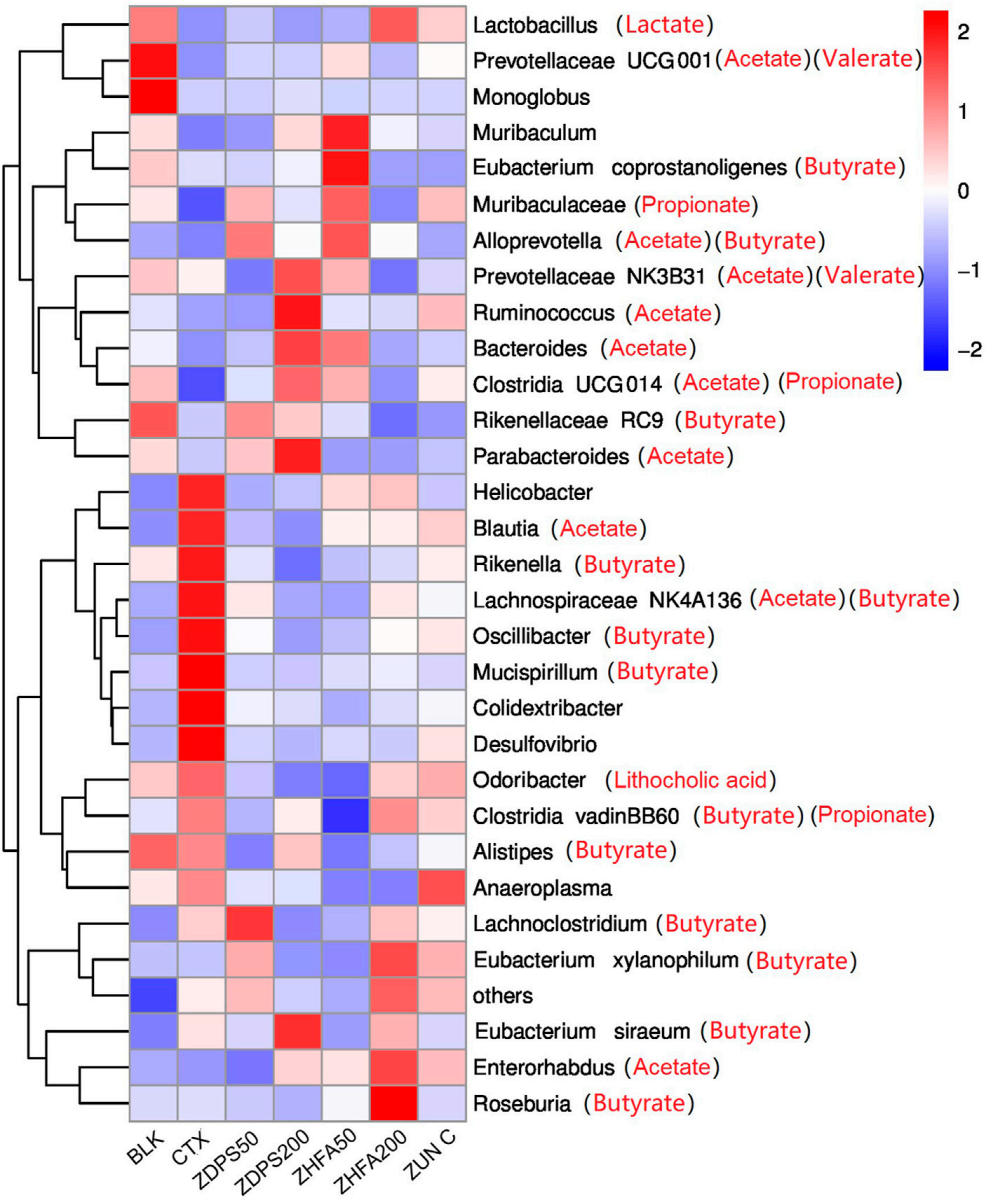
Effect of drugs on intestinal flora of mice ZUN C: zunyimyicn C; BLK: Blank control; CTX:Cyclophosphamide; ZDPS50: ZDPS 50 mg/kg/d; ZDPS200: ZDPS 200 mg/kg/d; ZHFA50: ZHFA 50 mg/kg/d; ZHFA200: ZHFA 50 mg/kg/d. The intestinal bacteria in the each group changed greatly at the genus level. The species abundance of the bacteria that accounted for the most of the total number of bacteria was arranged from high to lowthe community structure of different groups of mice varied at the genus level. Compared with CTX model mice, change of relative abundance of intestinal flora higher than 1 was defined as upregulation and lower than −1 as downregulation. Short chain fatty acids produced by the strain are shown in brackets.

The abundance of intestinal flora of animals in each group affected by drugs was ranked at the taxonomic level of different species, such as phyla, class, order, family, genus, and species, and the influence of drugs on the structure and composition of intestinal flora was analyzed according to their abundance changes. Figure 8 shows the ranking results of the top 30 species at the phyla and species levels. The species with the highest abundance in all treatment groups were classified into the eight main microflora and only a few other microflora species (others).

At the classification level of phyla (Figure 7A), the dominant intestinal microflora of BLK mice without drug treatment were mainly Bacteroidota, Firmicutes, Actinobaciota, Campilobacterota, Proteobateria, Desulfobacterota, and Pattescibateria, whereas Deferribacterota was not detected. After being treated with the drug CTX, the order of dominant bacteria was Bacteroidota, Firmicutes, Campilobacterota, Desulfobacterota, Deferribacterota, Actinobaciota, Proteobateria, and Pattescibacteria. Among them, the abundance of Bacteroidota decreased significantly, whereas the abundance of Firmicutes, Campilobacterota, and Desulfobacterota increased significantly. After the intervention of the drug ZDPS50, the order of dominant bacteria was Bacteroidota, Firmicutes, Campilobacterota, Actinobaciota, Proteobateria, Desulfobacterota, Deferribacterota, and Pattescibateria. The abundance of Bacteroidota and Proteobateria increased, whereas the abundance of Firmicutes and Campilobacterota decreased. After the intervention of the drug ZDPS200, the abundance of Bacteroidota and Actinobaciota further increased, while Firmicutes decreased. However, the extent of the decrease in Campilobacterota abundance was not as large as that in the intervention group of the drug ZDPS50, while Deferribacterota and Pattescibacteria were completely suppressed to a level that could not be detected. The overall change trend of the flora abundance after the intervention of the drug ZHFA50 was similar to that of the drug ZDPS200, but the abundance of Bacteroidota remarkably increased, and the abundance of Firmicute decreased. The abundance of Campilobacterota was higher than in the intervention groups ZDPS200 and ZDPS50. Notably, the high concentration of ZHFA200 drug intervention showed different effects from ZHFA50, and the order of abundance of the dominant bacteria in the ZHFA200 drug intervention group was Firmicutes, Bacteroidota, Campilobacterota, Actinobaciota, Desulfobacterota, Deferribacterota, Proteobateria, and Patescibacteria. In other words, ZHFA200 drug significantly decreased the abundance of Bacteroidota and Campilobacterota while increasing the abundance of Firmicutes and Actinobaciota. For the mice treated with the new antibiotic zunyimycin, the order of the abundance of intestinal flora is Bacteroidota, Firmicutes, Campilobacterota, Actinobaciota, Desulfobacterota, Proteobateria, and Deferribacterota, while the abundance of Pattescibateria decreased to a level that cannot be detected. In short, zunyimycin C increases the abundance of Bacteroidota, Actinobaciota, Desulfobacterota, Proteobacteria, Deferribacterota.

The effects of drugs on the structure and composition of intestinal flora at the species level are complex (Figure 7B). In the BLK group mice without drug treatment, the top 30 most abundant intestinal flora at the level of species were *B acidifaciens*, *L. murinus*, *C. bacterium*, *L. reuteri*, *L. intestinalis*, *L. bacterium*, *B. caecimuris*, *P. gordonii*, *B. stercorirosoris*, *B. bacterium*, *Eubacterium* sp., *E. coli*, *Dorea* sp., *C. stationis*, *A. Colihominis*, *Clostridium* sp., *C inhibens*, *A. lwoffii*, *Clostridium* sp., *B. vulgatus*, *A. stercorihominis*, *A. muris*, *A. urinaeequi*, and *H. hepaticus*. The abundance of *finegoldii* and other species cannot be detected. However, after the use of CTX drugs to reduce animal immunity, the abundance of its species changed remarkably, and the order of dominant bacteria abundance is *C. bacterium*, *B. acidifaciens*, *L. bacterium*, *L. johnsonii*, *P. goldsteinii*, *L. intestinalis*, *L. reuteri*, *P. gordonii*, *P. distasonis*, *L. murinus*, *B. caecimuris*, *L. bacterium*, *B. stercorirosoris*, *B. bacterium*, *A. colihominis*, *Clostridium* sp*.*, *A. finegoldii*, *E. coli*, *B. vulgatus*, and *Eubacterium* sp. The abundance of muris decreased to an undetectable level. After the intervention of ZDPS50, the abundance of intestinal flora in mice changed, and the abundance of dominant flora is in the order of *B acidifaciens*, *L. johnsonii*, *Lactobacillus*, *C. bacterium*, *B. caecimuris*, *L. murinus*, *L. reuteri*, *L. intestinalisus*, *B. bacterium*, *P. goldsteinii*, *P. gordonii*, *L. bacterium, Dorea* sp., *C. stationis*, *A. stercorihominis*, *A. colihominis*, *E. colia-Shigella*. And *P. distasonis*, *L. bacterium*, *B. stercorirosoris*, *Clostridium* sp., *A. finegoldii*, *B. vulgatus*, *Eubacterium* sp., *Lachnospiraceae* FCS020, *C. inhibens*, *A. lwoffii*, *Clostridium* sp., *A. muris*, and *A. urinaeequi.* The abundance of hepaticus decreased to an undetectable level. Similarly, after the intervention of ZDPS200, the abundance of intestinal flora in mice changed, and the abundance of dominant flora is in the order of *B. acidifaciens*, *L. murinus*, *P. gordonii*, *P. goldsteinii*, *B. caecimuris*, *B. stercorirosoris*, *C. bacterium*, *Clostridium* sp., *E. coli*, *H. hepaticus*, *A. colihominis*, *L. reuteri*, *B. bacterium*, and *L. bacterium*. The abundance of urinaeequi decreased to an undetectable level. After the intervention of ZHFA50, the diversity of intestinal flora in mice was further reduced, and abundance of flora was changed, and the abundance of dominant flora were *B. acidifaciens*, *L. johnsonii*, *L. reuteri*, *L. murinus*, *L. bacterium*, *P. gordonii*, *P. distasonis*, *Eubacterium* sp., *P. goldsteinii*, *L. bacterium*, *L. intestinalis*, *A. colihominis*, *B. vulgatus*, and *E. coli*. However, the abundance of *B. caecuris*, *B. stercorosoris*, *C. bacterium*, *Clostridium* sp., *H. hepaticus*, *Dora* sp., *C. stationis*, *A. stercorihominis*, *Clostridium* sp., *A. finegoldii*, *Lachnospirace* FCS020. *C. inhibens*, *A. lwoffii*, *A. muris* and *A. urinaequi* decreased to an undetectable level. After the intervention of ZHFA200, the diversity of intestinal flora in mice was further reduced, the flora abundance was changed, and the dominant flora abundance was in the order of *L murinus*, *L. johnsonii*, *B. acidifaciens*, *L. reuteri*, *L. intestinalis*, *L. bacterium*, *B. caecimuris*, *Eubacterium* sp., *L. bacterium*, *C. bacterium*, *P. gordonii*, *C. stationis*, *Dorea* sp., *P. goldsteinii*, *E. coli*, *A. lwoffii*, *B. vulgatus*, and *A. colihominis*. However, the abundance of *P. distasonis*, *B. bacterium*, *B. stereosoris*, *Clostridium* sp., *H. hyperpaticus*, *A. stereohominis*, *Clostridium* sp., *A. finegoldii*, *Lachnospirace* FCS020, *C. inhibens*, *A. muris* and *A. urinaequi* decreased to an undetectable level. After the intervention of antibiotic zunyimycin, the diversity of intestinal flora in mice was further reduced, the abundance of flora was changed, and the abundance of dominant flora was in the order of *B. acidifaciens*, *L. johnsonii*, *L. intestinalis*, *L. murinus*, *L. reuteri*, *L. bacterium*, *B. caecimuris*, *P. goldsteinii*, *P. gordonii*, *Lachnospiraceae* FCS020, *C. inhibens*, *Clostridium* sp., *E. coli*, *Escherichia-Shigella*, *A. lwoffii*, *A. urinaeequi*, and *A. stercorihominis*. However, the abundance of *Eubacterium* sp., *L. bacterium*, *C. bacterium*, *C. stationis*, *Dora* sp., *B. vulgatus*, *A. colihominis*, *P. distasonis*, *B. bacterium*, *B. stereosoris*, *Clostridium* sp., *H. hepaticus*, *A. finegoldii*, and *A. muris* decreased to an undetectable level.

## 4 Discussion

*Phellinus igniarius* is a traditional fungus with the same origin of medicine and food, and it is widely distributed around the world. *P. igniarius* has long been used for clinical application and basic research to improve the immunity of the body (Zhao et al., 2019).

Phellinus polysaccharides activate humoral, immune regulation, and cellular immune regulation and promote the body to release a large amount of immune stimulating products (Li et al., 2014). Since then, a large number of studies have found that Phellinus has anti-inflammatory, analgesic, anti-cancer, antibacterial, anti-fibrosis, and antioxidant effects (Kim et al., 2019).

Habitat and parasitic plants remarkably affect the production of active species of traditional Chinese medicine fungi (Jan et al., 2022). The *P. igniarius* YASH1 species used in the present study is a wild locust tree collected from the mountaintop of the hinterland of the Loess Plateau at an altitude of 1,100 m. This area has a natural climate of drought, strong sunlight, and high ultraviolet radiation that differs from other regions in the world and is a special ecological environment of the Loess Plateau.

The active substances produced by *P. igniarius* YASH1 parasitized on locust trees in northern Shaanxi may be quite different from those produced by the same kind of fungi in other regions.

The monosaccharide composition and proportion of polysaccharides obtained from different habitats of *P. igniarius* were compared and analyzed, and results showed that the polysaccharides from *P. igniarius* YASH1 were different from those from other reported habitats. Only a small amount of polysaccharides from the mulberry fungus may contain rhamnose monosaccharide (Table 4), for example, the monosaccharide of ZDPS is mannose: rhamnose: glucose: galactose:xylose:arabinose:fucose = 0.211:0.0139:1:0.162:0.037:0.027:0.1236, while the monosaccharide of JDPS is rhamnose: glucose: galactose: xylose: arabinose: fucose = 0.17:0.0106:1:0.46:0.028:0.0048:0.172. These two polysaccharides both have the unique monosaccharide component rhamnose, which is not possessed by most polysaccharides from other sources of *P. igniarius*, and their rhamnose and galactose content is higher than that of polysaccharides from other sources of *P. igniarius*. Rhamnose and galactose, as important prebiotics, play an important role in intestinal flora, making them important for their unique immune regulation role (Li et al., 2015; Suabjakyong et al., 2015).

**TABLE 4:** Rhamnose containing fungal polysaccharides from *P. igniarius* fungi.

Polysaccharide	Monosaccharide composition	Source	References
ZDPS	Mannose: Rhamnose: Glucose: galactose: xylose: arabinose: Fhaloose = 0.211:0.0139:1:0.162:0.037:0.027:0.1236	sporophore	This work
JDPS	Rhamnose: Glucose: galactose: xylose: arabinose: Trehalose = 0.17:0.0106:1:0.46:0.028:0.0048:0.172	mycelium	This work
P1	Fucinose: rhamnose: Galactose: Glucose: xylose: mannose: 3-O-Me-d-galactose = 1:3.12:33.51:2.03:4.03:1.09:2.87	sporophore	Li et al. (2011)
PM-ESP1	Rhamnose: galactose: Glucose: mannose: glucuronic acid = 3.5:3.3:12.5:76.3:4.6	mycelium	Cao et al. (2013)
PM-ESP3	Rhamnose: galactose: glucose: mannose: glucuronic acid = 5.8:1.0:2.4:7.3:81.2	mycelium	Cao et al. (2013)
PIPS	Glucose: rhamnose: mannose = 11.0:14.0:1.0	mycelium	Zhang et al. (2014)
C-PIPS	Glucose: rhamnose: mannose = 9.0:3.0:1.0	mycelium	Zhang et al. (2014)
IPSW-4	Rhamnose: Xylose: Mannose: Glucose: galactose = 1.29:1.21:1:43.86:1.86	mycelium	Li et al. (2015)
SP-50	Rhamnose: arabinose: galactose: Glucose: xylose: mannose: galacturonic acid: glucuronic acid = 11.57:57.24:54.25:32.95:65.52:49.44:19.09:8.25	mycelium	Li et al. (2015)
PLP	Rhamnose: Mannose: arabinose: Galactose: xylose: Glucose = 0.82:8.32:1.13:8.06:2.80:78.88	sporophore	Suabjakyong et al. (2015)
PIP	Rhamnose: Mannose: arabinose: Galactose: xylose: Glucose = 1.31:14.51:2.63:20.65:3.32:57.58	sporophore	Suabjakyong et al. (2015)
EPS-Glc	Xylose: Glucuronic acid: galactose: Glucose: mannose: galacturonic acid = 1.52:19.53:2.39:24.28:30.68:21.60	mycelium	Xu et al. (2017)
EPS-Fru	Rhamnose: ribose: xylose: glucuronic acid: galactose: Glucose: mannose: galactose, uronic acid = 1.42:1.22:2.05:35.87:2.77:7.77:42.27:6.63	mycelium	Xu et al. (2017)
JZx	Glucose: mannose: rhamnose = 2.0:1.0:16.0	mycelium	Zhang et al., 2018
CK	Glucose: mannose: rhamnose = 9.0:1.0:3.0	mycelium	Zhang et al., 2018
IHSFP	Glucose: Rhamnose: arabinose: Mannose: galactose: Glucose N = 96.19:0.53:0.73:0.56:1.75:0.25	mycelium	Liu et al. (2019)
SVP	Mannose: rhamnose: glucurononic acid: galacturonic acid: glucose N: glucose: galactose N: galactose:xylose: arabinose: fucinose = 1.63:0.04:0.36:0.03:0.13:8.39:0.08:1.08:0.25:1.07:0.40	sporophore	Wan et al. (2020)

In comparison with other reported flavonoids from Phellinus, the flavonoids from *P. igniarius* YASH1 parasitized on wild Sophora japonica in northern Shaanxi also have their own unique composition and content differences. For example, the composition and content of the total flavonoid ZHFA in the sporophore are glycyrrhizin (581 ng/mL), cyanidin 2 (243 ng/mL), dihydroquercetin-7-0-rhamnoglycan (161 ng/mL), protocatechuic aldehyde 1 (147 ng/mL), p-coumaric acid (130 ng/mL) protocatechuic acid 2 (124 ng/mL), ethyl vanillin 2 (102 ng/mL), myricetin 1 (93.9 ng/mL), p-coumaric acid 1 (73 ng/mL), phenylalanine 1 (64.4 ng/mL), proanthocyanidin B1 (47.2 ng/mL), caffeic acid 1 (38.3 ng/mL), resveratrol glycoside 2 (35.8 ng/mL), vanillin 1 (31.4 ng/mL), apigenin 1 (30.7 ng/mL), proanthocyanidin B2 (24.7 ng/mL), apigenin 2 (5.66 ng/mL), glycyrrhizic acid 1 (4.53 ng/mL) resveratrol glycerol 1 (4.15 ng/mL), and vanillin 1 (0.381 ng/mL). However, the diversity of total flavonoids in its mycelia is much reduced, and its main components are quercetin 1 (143 ng/mL), p-coumaric acid 2 (122 ng/mL), 2,4,6-tribromophenol 2 (108 ng/mL), caffeic acid 1 (103 ng/mL), protocatechuic acid 2 (98.1 ng/mL), myricetin 1 (95 ng/mL), ethyl vanillin 2 (90.5 ng/mL), phenylalanine 1 (47.4 ng/mL), resveratrol glycoside 2 (37.6 ng/mL), apigenin 1 (34.7 ng/mL), and vanillin 1 (29.4 ng/mL), resveratrol glycol 1 (24 ng/mL), protocatechuic aldehyde 1 (15.4 ng/mL), vanillin 1 (14.2 ng/mL), trans resveratrol 2 (13.9 ng/mL), vanillin 2 (8.2 ng/mL), and apigenin 2 (2.49 ng/mL). This result also well reflects the scientific principle that the differences in geographical environment and parasitic host plants lead to the differences in the active products of wild *P. igniarius*, and the cultivation methods have an important effect on the types and quantities of its active products (Thanh et al., 2018). Based on the recognized basic theory of “targeted BGC discovery strategies” developed by active natural products (O'Neill et al., 2016; Kalkreuter et al., 2020), ZPDS/JDPS and ZHFA/JHFA have their own unique biological activity or activity mechanism caused by the differences between their own basic chemical structural units or composition and the known polysaccharides or flavonoids from *P. igniarius*. In antioxidant and hydroxyl radical scavenging capacity assay (Figure 1), it is interesting that the hydroxyl radical scavenging activity between wild-type and laboratory-type polysaccharides and flavonoids is very different in the level of concentration (even 10 times). The differences in the chemical composition of polysaccharides or flavonoids may be an important reason for this huge activity difference. It is also suggested that some particular monosaccharide fractions or flavonoids in our study may have important effects on the activities of polysaccharides or total flavonoids and may be of some reference value in the search for novel antioxidants.

This work also confirmed the antioxidant activity and free radical scavenging activity of ZPDS/JDPS and ZHFA/JHFA at the chemical level, and the cell experimental results support the immunoenhancement activity of these two kinds of drugs. The four extracts ZPDS/JDPS and ZHFA/JHFA can promote the proliferation of immune cells and the change of cell morphology. After being stimulated with drugs, the size of Jurkat cell clusters remarkably increased, and they became more dispersed. The number of macrophages increased significantly, the cell polarization enhanced the phagocytic function of macrophages along with the increase in the number of Raji cells, and cell aggregation became more obvious. In mice intervened with ZDPS and ZHFA, the levels of IL-2, IL-6, and IFN-γ recovered when compared to the CTX group. However, ZHFA did not increase the level of TNF-α, indicating that ZHFA may slightly inhibit TNF-α, and ZHFA may slightly inhibit TNF-α generation. ZHFA may not rely on TNF-α to enhance immunity. In comparison with the CTX group, the thymus index and spleen index of immune organs such as the thymus and spleen of mice intervened by drugs ZDPS and ZHFA significantly increased. At the same time, the dose of the drug did not significantly change the indicators of other organs in mice. Therefore, the drug has no toxicity or side effects on the body’s immune system within a certain dose range. The changes in the levels of cytokines IL-2, IL-6, IFN-γ, and TNF-α are closely related to the body’s immune status. ZDPS can increase IL-2, IL-6, and TNF-α but decrease IFN-γ at the cellular level (Figure 4). However, Both low and high doses of ZDPS upregulated the expression of IL-2, IL-6, and TNF-α, but affected IFN-γ at the animal level. Flavone ZHFA showed similar activity to ZDPS in terms of regulating cytokine expression at the cellular level, but at the animal level, both low and high doses of ZHFA increased the expression of IL-2 and IL-6 in mice but decreased TNF-α.

The homeostasis of the intestinal flora can directly affect the changes in the level of cytokine expression in the body (Bullard et al., 2022). The changes in the homeostasis of intestinal flora can be reflected in the composition and abundance of the flora and the changes in the metabolic product SCFAs of intestinal flora (Ashique et al., 2022). The changes in the seven major SFCAs in mouse feces, such as acetate, butyrate, caprate, isobutyrate, isovalerate, valerate, and propionate, before and after drug intervention were investigated. The drug CTX significantly reduced the content of these SCFAs in the intestinal tract, while different drug interventions had different effects. Different doses of drugs such as ZDPS, ZHFA, and zunyimycin can promote the recovery of acetate and propionate in the intestine, but all drugs further reduced the content of caproate in the intestine, even to the extent that cannot be detected. Surprisingly, in addition to the high concentration of ZHFA can further reduce the concentration of butyrate. For low-dose ZDPS, the drug can promote the recovery of isobutyrate, isovalerate, and valerate, but high-dose ZDPS can reduce the concentration of valerate. Aside from promoting the production of isobutyrate and isovalerate, low dose of ZHFA50 plays a role in reducing the production of the two other SCFAs, namely, caprate and valerate. The effects of high concentration of ZHFA and zunyimicin are basically the same. Both drugs can increase the content of acetate and propionate in the intestine, but they can both reduce the content of the five other SCFAs. Considering that acetic acid, propionic acid, and butyric acid account for more than 90% of the total SCFA content in the intestine, acetic acid and butyric acid can affect intestinal health, have anti-inflammatory effects, regulate immune cells, and restore immune function. However, in the present study, except for the high concentration of ZHFA, other drugs reduced the concentration of butyrate in the intestine, and the mechanism of this is worthy of further discussion. At the species level, the changes of the highest abundance of TOP30 bacteria were analyzed in animals treated with different drugs (Figure 8). After the mice were treated with CTX, the relative abundance of at least five bacteria responsible for the synthesis of acetate and butyrate in their intestinal flora was highest possibly because of the body mobilizing immune regulation and the attempt to restore the normal state. The highest relative abundance is also related to the synthesis of acetate and butyrate.

## 5 Conclusion

Habitat and parasitic host plants can affect the monosaccharide composition of *P. igniarius* polysaccharides and the chemical composition of its flavonoids. The polysaccharides and flavonoids of *P. igniarius* YASH1 are different from the reported chemical composition of polysaccharides and flavonoids from *P. fungi*. Polysaccharides and flavonoids from *P. igniarius* YASH have the ability of antioxidation and scavenging oxygen free radicals *in vitro* and can affect the expression of various cytokines *in vitro*. Polysaccharide ZDPS can play an immunomodulatory role by affecting the proliferation of immune cells and the level of cytokines when used in low doses. The drug range can improve the spleen and thymus index of animals in a certain concentration but have little effect on other organs, which has certain drug safety. Both polysaccharide ZDPS and flavone ZHFA can affect the output of SCFA by affecting the relative abundance of the bacteria responsible for the synthesis of SCFA in intestinal flora, but the mechanisms involved need further research. In conclusion, the polysaccharide of *P. igniarius* YASH from special habitats and parasitic species can enhance the immunity of immunocompromised mice caused by CTX, which has great clinical application potential and is worth further research and development.

## Data Availability

The datasets presented in this study can be found in online repositories. The names of the repository/repositories and accession number(s) can be found in the article/Supplementary Material.

## Supplementary material

SUPPLEMENTARY FIGURE S1 Effect of drugs on the recovery of organ index in mice. **(A)** Thymus index. **(B)** Spleen index. **(C)** Liver index. *: significant difference (*p* < 0.05); **: significant difference (*p* < 0.01); ***: significant difference (*p* < 0.001).

## References

[B1] Ashique S. De Rubis G. Sirohi E. Mishra N. Rihan M. Garg A. (2022). Short Chain Fatty Acids: Fundamental mediators of the gut-lung axis and their involvement in pulmonary diseases. Chem. Biol. Interact. 368, 110231. 10.1016/j.cbi.2022.110231 36288778

[B2] Avci E. Akarslan Z. Z. Erten H. Coskun-Cevher S. (2014). Oxidative stress and cellular immunity in patients with recurrent aphthous ulcers. Braz J. Med. Biol. Res. 47 (5), 355–360. 10.1590/1414-431x20143714 24760117 PMC4075302

[B3] Bullard B. M. VanderVeen B. N. McDonald S. J. Cardaci T. D. Murphy E. A. (2022). Cross talk between the gut microbiome and host immune response in ulcerative colitis: Nonpharmacological strategies to improve homeostasis. Am. J. Physiol. Gastrointest. Liver Physiol. 323 (6), G554–G561. 10.1152/ajpgi.00210.2022 36283090 PMC9678428

[B4] Cao C. Peng F. Cui B. K. (2013). Chemical characterization and structure of exopolysaccharides from submerged culture of new medicinal mushroom from China, Phellinus mori (higher Basidiomycetes). Int. J. Med. Mushrooms 15 (1), 57–69. 10.1615/intjmedmushr.v15.i1.70 23510285

[B5] Chen H. Tian T. Miao H. Zhao Y. Y. (2016). Traditional uses, fermentation, phytochemistry and pharmacology of *Phellinus linteus*: A review. Fitoterapia 113, 6–26. 10.1016/j.fitote.2016.06.009 27343366

[B6] Chen Y. Yao F. Ming K. Wang D. Hu Y. Liu J. (2016). Polysaccharides from traditional Chinese medicines: Extraction, purification, modification, and biological activity. Molecules 21 (12), 1705. 10.3390/molecules21121705 27983593 PMC6273901

[B7] Chopra H. Mishra A. K. Baig A. A. Mohanta T. K. Mohanta Y. K. Baek K. H. (2021). Narrative review: Bioactive potential of various mushrooms as the treasure of versatile therapeutic natural product. J. Fungi (Basel). 7 (9), 728. 10.3390/jof7090728 34575766 PMC8466349

[B8] Chugh R. M. Mittal P. Mp N. Arora T. Bhattacharya T. Chopra H. (2022). Fungal mushrooms: A natural compound with therapeutic applications. Front. Pharmacol. 13, 925387. 10.3389/fphar.2022.925387 35910346 PMC9328747

[B9] Djaldetti M. Bessler H. (2015). High temperature affects the phagocytic activity of human peripheral blood mononuclear cells. Scand. J. Clin. Lab. Invest. 75 (6), 482–486. 10.3109/00365513.2015.1052550 26067609

[B10] Gao W. Wang W. Sun W. Wang M. Zhang N. Yu S. (2017). Antitumor and immunomodulating activities of six Phellinus igniarius polysaccharides of different origins. Exp. Ther. Med. 14 (5), 4627–4632. 10.3892/etm.2017.5191 29109758 PMC5663028

[B11] Hu Q. Wu C. Yu J. Luo J. Peng X. (2022). Angelica sinensis polysaccharide improves rheumatoid arthritis by modifying the expression of intestinal Cldn5, Slit3 and Rgs18 through gut microbiota. Int. J. Biol. Macromol. 209, 153–161. 10.1016/j.ijbiomac.2022.03.090 35318077

[B12] Jan M. Mir T. A. Khare R. K. Saini N. (2022). “Adaptation strategies of medicinal plants in response to environmental stresses,” in Environmental challenges and medicinal plants. Environmental challenges and solutions. 2022. Editor Aftab T. (Cham: Springer).

[B13] Kalkreuter E. Pan G. Cepeda A. J. Shen B. (2020). Targeting bacterial genomes for natural product discovery. Trends Pharmacol. Sci. 41 (1), 13–26. 10.1016/j.tips.2019.11.002 31822352 PMC6938545

[B14] Kim E. H. Choi Y. S. Kim Y. M. (2019). Antioxidative and anti-inflammatory effect of Phellinus igniarius on RAW 264.7 macrophage cells. J. Exerc Rehabil. 15 (1), 2–7. 10.12965/jer.1938010.005 30899728 PMC6416490

[B15] Kong X. Hellermann G. R. Patton G. Kumar M. Behera A. Randall T. S. (2005). An immunocompromised BALB/c mouse model for respiratory syncytial virus infection. Virol. J. 2, 3. 10.1186/1743-422X-2-3 15701174 PMC549044

[B16] Li L. Wu G. Choi B. Y. Jang B. G. Kim J. H. Sung G. H. (2014). A mushroom extract Piwep from Phellinus igniarius ameliorates experimental autoimmune encephalomyelitis by inhibiting immune cell infiltration in the spinal cord. Biomed. Res. Int. 2014, 218274. 10.1155/2014/218274 24592383 PMC3922003

[B17] Li S. C. Yang X. M. Ma H. L. Yan J. K. Guo D. Z. (2015). Purification, characterization and antitumor activity of polysaccharides extracted from Phellinus igniarius mycelia. Carbohydr. Polym. 133, 24–30. 10.1016/j.carbpol.2015.07.013 26344250

[B18] Li X. Chu F. J. Jiang S. L. Jin X. B. Wang H. Liu Y. (2021). RNF144A-AS1, a TGF-β1- and hypoxia-inducible gene that promotes tumor metastasis and proliferation via targeting the miR-30c-2-3p/LOX axis in gastric cancer. Zhongguo Zhong Yao Za Zhi 46 (1), 177–182. 10.1186/s13578-021-00689-z 34583752 PMC8480077

[B19] Li X. Q. Yue C. W. Xu W. H. Lü Y. H. Huang Y. J. Tian P. (2020). A milbemycin compound isolated from Streptomyces Sp. FJS31-2 with cytotoxicity and reversal of cisplatin resistance activity in A549/DDP cells. Biomed. Pharmacother. 128, 110322. 10.1016/j.biopha.2020.110322 32505822

[B20] Li Y. G. Ji D. F. Zhong S. Zhu J. X. Chen S. Hu G. Y. (2011). Anti-tumor effects of proteoglycan from Phellinus linteus by immunomodulating and inhibiting Reg IV/EGFR/Akt signaling pathway in colorectal carcinoma. Int. J. Biol. Macromol. 48 (3), 511–517. 10.1016/j.ijbiomac.2011.01.014 21262260

[B21] Liebisch G. Ecker J. Roth S. Schweizer S. Öttl V. Schött H. F. (2019). Quantification of fecal short chain fatty acids by liquid chromatography tandem mass spectrometry-investigation of pre-analytic stability. Biomolecules 9 (4), 121. 10.3390/biom9040121 30925749 PMC6523859

[B22] Liu X. Hou R. Xu K. Chen L. Wu X. Lin W. (2019). Extraction, characterization and antioxidant activity analysis of the polysaccharide from the solid-state fermentation substrate of Inonotus hispidus. Int. J. Biol. Macromol. 123, 468–476. 10.1016/j.ijbiomac.2018.11.069 30445081

[B23] Lyngsie G. Krumina L. Tunlid A. Persson P. (2018). Generation of hydroxyl radicals from reactions between a dimethoxyhydroquinone and iron oxide nanoparticles. Sci. Rep. 8 (1), 10834. 10.1038/s41598-018-29075-5 30018415 PMC6050337

[B24] Madariaga V. I. Jasim H. Ghafouri B. Ernberg M. (2021). Myogenous temporomandibular disorders and salivary markers of oxidative stress-A cross-sectional study. J. Oral Rehabil. 48 (1), 1–9. 10.1111/joor.13100 32979853 PMC7820944

[B25] O'Neill E. C. Saalbach G. Field R. A. (2016). Gene discovery for synthetic biology: Exploring the novel natural product biosynthetic capacity of eukaryotic microalgae. Methods Enzymol. 576, 99–120. 10.1016/bs.mie.2016.03.005 27480684

[B26] Shi L. Tan Y. Sun Z. Ren A. Zhu J. Zhao M. (2019). Exogenous salicylic acid (SA) promotes the accumulation of biomass and flavonoid content in Phellinus igniarius (agaricomycetes). Int. J. Med. Mushrooms 21 (10), 955–963. 10.1615/IntJMedMushrooms.2019032557 32450033

[B27] Shi X. Liu D. Zhang J. Hu P. Shen W. Fan B. (2016). Extraction and purification of total flavonoids from pine needles of Cedrus deodara contribute to anti-tumor *in vitro* . BMC Complement. Altern. Med. 16, 245. 10.1186/s12906-016-1249-z 27461104 PMC4962484

[B28] Suabjakyong P. Nishimura K. Toida T. Van Griensven L. J. (2015). Structural characterization and immunomodulatory effects of polysaccharides from Phellinus linteus and Phellinus igniarius on the IL-6/IL-10 cytokine balance of the mouse macrophage cell lines (RAW 264.7). Food Funct. 6 (8), 2834–2844. 10.1039/c5fo00491h 26190688

[B29] Sułkowska-Ziaja K. Balik M. Muszyńska B. (2021). Selected species of the genus Phellinus - chemical composition, biological activity, and medicinal applications. Chem. Biodivers. 18 (11), e2100609. 10.1002/cbdv.202100609 34705323

[B30] Sun Y. Zhong S. Yu J. Zhu J. Ji D. Hu G. (2018). The aqueous extract of Phellinus igniarius (SH) ameliorates dextran sodium sulfate-induced colitis in C57BL/6 mice. PLoS One 13 (10), e0205007. 10.1371/journal.pone.0205007 30289941 PMC6173430

[B31] Thanh N. T. Tuan N. N. Kuo P. C. Dung D. M. Phuong D. L. Giang D. T. T. (2018). Chemical constituents from the fruiting bodies of Phellinus igniarius. Nat. Prod. Res. 32 (20), 2392–2397. 10.1080/14786419.2017.1413572 29232973

[B32] Wan X. Jin X. Xie M. Liu J. Gontcharov A. A. Wang H. (2020). Characterization of a polysaccharide from Sanghuangporus vaninii and its antitumor regulation via activation of the p53 signaling pathway in breast cancer MCF-7 cells. Int. J. Biol. Macromol. 163, 865–877. 10.1016/j.ijbiomac.2020.06.279 32629056

[B33] Wang X. Li Z. Sun R. Li X. Guo R. Cui X. (2022). Zunyimycin C enhances immunity and improves cognitive impairment and its mechanism. Front. Cell. Infect. Microbiol. 12, 1081243. 10.3389/fcimb.2022.1081243 36579344 PMC9791046

[B34] Wang Y. Q. Mao J. B. Zhou M. Q. Jin Y. W. Lou C. H. Dong Y. (2019). Polysaccharide from Phellinus Igniarius activates TLR4-mediated signaling pathways in macrophages and shows immune adjuvant activity in mice. Int. J. Biol. Macromol. 123, 157–166. 10.1016/j.ijbiomac.2018.11.066 30439422

[B35] Xu C. Yu J. Zhao S. Wu S. He P. Jia X. (2017). Effect of carbon source on production, characterization and bioactivity of exopolysaccharide produced by Phellinus vaninii Ljup. Acad Bras Cienc 89 (3), 2033–2041. 10.1590/0001-3765201720150786 29044312

[B36] Yuan Q. Zhao L. Li Z. Harqin C. Peng Y. Liu J. (2018). Physicochemical analysis, structural elucidation and bioactivities of a high-molecular-weight polysaccharide from Phellinus igniarius mycelia. Int. J. Biol. Macromol. 120, 1855–1864. 10.1016/j.ijbiomac.2018.09.192 30287368

[B37] Zapora E. Wolkowycki M. Bakier S. Zjawiony J. K. (2016). Phellinus igniarius: A pharmacologically active polypore mushroom. Nat. Prod. Commun. 11 (7), 1934578X1601100–1046. 10.1177/1934578x1601100741 30452190

[B38] Zhang H. Ma H. Liu W. Pei J. Wang Z. Zhou H. (2014). Ultrasound enhanced production and antioxidant activity of polysaccharides from mycelial fermentation of Phellinus igniarius. Carbohydr. Polym. 113, 380–387. 10.1016/j.carbpol.2014.07.027 25256498

[B39] Zhou X. Shi Q. Li J. Quan S. Zhang X. Gu L. (2022). Medicinal fungus Phellinus igniarius alleviates gout *in vitro* by modulating TLR4/NF-kB/NLRP3 signaling. Front. Pharmacol. 13, 1011406. 10.3389/fphar.2022.1011406 36339594 PMC9634182

