# Supplementary figures and images for: Immune-enhancing activity of polysaccharides and flavonoids derived from Phellinus igniarius YASH1
Source: Front Pharmacol. 2023 Apr 25;14:1124607. doi: 10.3389/fphar.2023.1124607 (PMC10166811; doi:10.3389/fphar.2023.1124607)

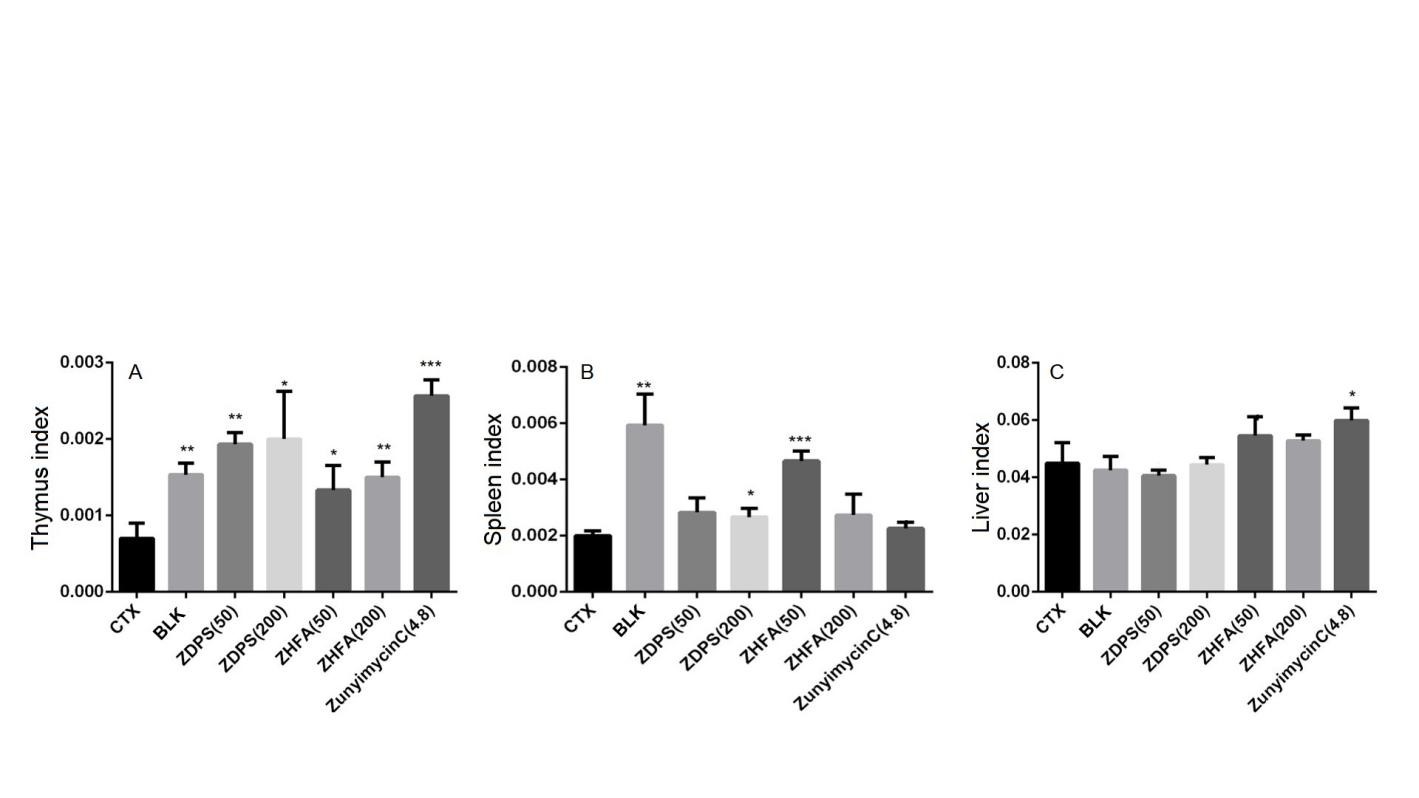

Supplement: Supplementary file 1 [file Image1.jpeg]
